# Functional Diversity of Nectary Structure and Nectar Composition in the Genus *Fritillaria* (Liliaceae)

**DOI:** 10.3389/fpls.2018.01246

**Published:** 2018-09-24

**Authors:** Katarzyna Roguz, Andrzej Bajguz, Agnieszka Gołębiewska, Magdalena Chmur, Laurence Hill, Paweł Kalinowski, Jürg Schönenberger, Małgorzata Stpiczyńska, Marcin Zych

**Affiliations:** ^1^Botanic Garden, Faculty of Biology, University of Warsaw, Warsaw, Poland; ^2^Department of Plant Biochemistry and Toxicology, Faculty of Biology and Chemistry, Institute of Biology, University of Bialystok, Bialystok, Poland; ^3^Petersham Lodge, Richmond, United Kingdom; ^4^Department of Nature Protection and Rural Landscape, Institute of Technology and Life Sciences, Falenty, Poland; ^5^Department of Botany and Biodiversity Research, University of Vienna, Vienna, Austria

**Keywords:** *Fritillaria*, nectar, pollinator shift, ornithogamy, nectary surface, SEM

## Abstract

*Fritillaria* is a genus consisting of 130 to 140 species of bulbous plants, native to temperate regions of the northern hemisphere. Generally viewed as an insect pollinated genus with the exception of two North American species, *Fritillaria gentneri* and *F. recurva*, which are described as hummingbird-pollinated and the Asian species, *F. imperialis*, described as passerine-pollinated. These pollinator shifts are possibly the result of adaptive changes to the structure and morphology of the nectary, as well as a change in the nectar concentration and composition. A study was conducted in a target group of 56 *Fritillaria* species, based on the morphology of their nectaries and nectar composition to assess the significance of pollination mode as well as its predisposition for the evolution of bird pollination. All species studied had nectaries located at their tepal base and produced nectar, but their size, shape, color, and composition all varied. Most fritillaries had hexose-rich nectar, in easily accessible and unprotected nectaries. Scanning electron microscope (SEM) analysis revealed that the surface of the nectaries of most *Fritillaria* species was flat and clearly distinct from that of the surrounding tissues, which might be regarded as an adaptation for insect-pollination. Nectaries of *F. imperialis* were considerably larger and had dilute nectar without sucrose, which was produced profusely, thereby fulfilling the criteria characteristic of ornithophilous flowers. The copious nectar of presumed hummingbird-pollinated species was rather balanced and of medium sugar concentration. Their large lanceolate nectaries contrasted sharply with the tessellated background of their tepals. These characters might indicate a mixed pollination system that engages both birds and insects. Floral anatomy and microstructure and nectar composition for *Fritillaria* species in subgenera *Korolkowia* and *Liliorhiza* are studied for the first time.

## Introduction

The genus *Fritillaria* L. (type species *F. meleagris* L.) is a widely distributed member of Liliaceae (lily family). It comprises of 130 to 140 species occurring through most of the northern hemisphere (Tamura, 1998; Rønsted et al., 2005; Tomović et al., 2007; Mabberley, 2008; Day et al., 2014), with centers of speciation in the Mediterranean region, especially in Turkey and Greece (Rix, 1984; Zaharof, 1986; Tekşen and Aytaç, 2011), Iran (Kiani et al., 2017), Western North America (Beetle, 1944; Rønsted et al., 2005), and East Asia (Hill, 2016). Recent phylogenetic analyses indicate that the genus may be paraphyletic with members of the subgenus *Liliorhiza*, principally North American species, forming a sister clade to the remaining *Fritillaria* species and the genus *Lilium* (Day et al., 2014).

*Fritillaria* are found in a variety of climatic regions and in different habitats, with about half of the genus occurring in locations categorized as open with dry summers (Rønsted et al., 2005). Fritillaries are located across a wide latitudinal range from coasts, riparian zones, meadows, woodland, steppe, deserts, mountain screes, and alpine zones (Xinqi and Mordak, 2000; Ness, 2003; Tekşen and Aytaç, 2011; Zych and Stpiczyńska, 2012; Rix and Strange, 2014).

*Fritillaria* species are bulbiferous, spring-flowering perennials with an erect flowering stem producing either a single flower or multi-flowered racemes. The flowers are usually actinomorphic and have a typical tulip-like, trimerous, campanulate perianth but with a nodding character (Tamura, 1998). They come in various colors such as white, pink, greenish, yellow, or purplish/reddish. The perianth parts of many species, including the type species, have a characteristic checkerboard pattern; hence, the name of the genus – fritillus, Latin for dicebox (Zych et al., 2014).

This astonishing floral diversity may have developed in response to their pollinator interactions, although, flower visitors have only been recorded for six species of *Fritillaria* (White, 1789; Hedström, 1983; Búrquez, 1989; Peters et al., 1995; Bernhardt, 1999; Minagi et al., 2005; Kawano et al., 2008; Zox and Gold, 2008; Zych and Stpiczyńska, 2012). These limited records are unlikely to reflect the complete spectrum of pollination vectors. Pollinator effectiveness has only been evaluated for bumblebee-pollinated *F. meleagris* (Zych and Stpiczyńska, 2012; Zych et al., 2013, 2014) and to a lesser extent the pollination of *F. imperialis* by birds and insects (Peters et al., 1995).

The first observation of a *Fritillaria* flower visitor was made by White (1789). He recorded small birds drinking nectar from *F. imperialis*. After 200 years, similar observations were also made for *F. imperialis* in other European gardens by Burques (1989) and Peters et al. (1995). Blue and great tits visited and efficiently pollinated flowers. Bumblebees were also seen visiting and pollinating the large and pendant flowers of *F. imperialis*, but pollinator efficiency was lower than bird visitations (Peters et al., 1995). Although there are no pollinator records for *F. imperialis* in the wild, it has been proposed that bird pollination is the most likely vector. This hypothesis is supported by the presence of a landing platform suitable for passerine birds and large pendant flowers with large volumes of diluted nectar (Peters et al., 1995). For some North American species (*F. gentneri* and *F. recurva*), hummingbird pollination has been recorded in natural habitats (Pendergrass and Robinson, 2005; Cronk and Ojeda, 2008), possibly attracted by their bright red flowers. The only *Fritillaria* species occurring in both Asia and North America, the typically dark-flowered *F. camschatcensis*, is habitually visited by flies (Zox and Gold, 2008). Flowers of this species emit a disagreeable smell like rotting flesh, and it might be described as a typical example of carrion fly-pollination syndrome (Willmer, 2011). There are only six fritillaries that have been noted to have a sweet scent: *F. liliacea* (King, 2001), *F. obliqua* (Beck, 1953), *F. striata* (Santana, 1984), *F. stribrnyi* (personal observation LH), *F. tortifolia* (personal observation LH), and *F. yuminensis* (Leon et al., 2009), all presumably visited by bee species. Bees have only been recorded to visit *F. ayakoana* (Minagi et al., 2005) and *F. meleagris* (Hedström, 1983; Stpiczyńska et al., 2012; Zych et al., 2014).

Generally, flower pollinators are attracted by a combination of visual and olfactory cues. Although the data is limited, one might assume this is true for *Fritillaria*. Floral scent, but to the best of our knowledge, it has only been explored in studies for *F. meleagris* (Hedström, 1983). In the study conducted by Zych and Stpiczyńska (2012), many bees collected *Fritillaria* pollen, but the most common floral food-reward appeared to be nectar. This was secreted by perigonal nectaries positioned adaxially on each of the six perianth segments (Stpiczyńska et al., 2012). Until now, the most comprehensive description of *Fritillaria* nectar diversity was given by Rix and Rast (1975), who studied nectar sugars in 37 European and Asiatic species of the genus. These authors found that nectar generally contained all three common nectar sugars, namely sucrose, glucose, and fructose. The only exception in their dataset was *F. imperialis* which produced no sucrose, confirming an earlier report by Wykes (1952). Rix and Rast (1975) concluded that nectar composition and, in particular, the fructose/glucose ratio may be an important diagnostic character in the infrageneric taxonomy of *Fritillaria*.

The position, morphology, and structure of nectar-secreting glands have been key taxonomic characters and have been investigated by many researchers, notably in Asiatic species by Bakhshi Khaniki and Persson (1997) and for *F. meleagris* by Stpiczyńska et al. (2012). For example, nectaries are lanceolate to linear in members of the subgenus *Fritillaria*, circular in the subgenus *Petilium* (L.) Baker, and in the subgenus *Rhinopetalum* (Fisch. ex Alexand) the nectaries are deeply depressed and situated in sac-like projections (Bakhshi Khaniki and Persson, 1997). This latter study, however, provides only general morphological descriptions and information concerning the ultrastructure of nectaries, is only available for *F. meleagris* (Stpiczyńska et al., 2012; Zych and Stpiczyńska, 2012). Studies of nectaries and nectar characteristics are important, not only from a taxonomic point of view but also with respect to ecological and evolutionary studies of the genus. For example, based on nectar characteristics, Rix and Rast (1975) posited that bees and wasps were the main pollinators of *Fritillaria*. In fact, floral visitors of *Fritillaria* flowers include insects of the orders Hymenoptera (mostly various species of bees and wasps), Diptera, Lepidoptera, and Coleoptera (Hedström, 1983; Bernhardt, 1999; Naruhashi et al., 2006; Kawano et al., 2008; Zych and Stpiczyńska, 2012; Zych et al., 2013). Although usually concealed by the perianth, in some *Fritillaria* species optically copious nectaries also play an important ecological role in guiding the pollinators to the nectar once they have been attracted to the flowers by other traits (tepal color, scent, etc.). This appears to be the case in ornithophilous *F. imperialis* (Cronk and Ojeda, 2008) and, perhaps, also in other bird-pollinated species. However, despite the diversity and wide distribution of the genus, these aspects of *Fritillaria* diversity are still neglected to a great extent. In particular, detailed studies of the flower, and, especially the study of nectar-secreting structures and nectar composition have only been conducted for a very limited number of species. To date, the most complete analysis using light microscopy (LM) and scanning electron microscopy (SEM) was completed by Bakhshi Khaniki and Persson (1997) for members of four subgenera represented in Central Asia: *Fritillaria*, *Petilium* (L.) Baker, *Rhinopetalum* (Fisch. ex Alexand) Baker, and *Theresia* (K. Koch) Baker. However, no information is available for fritillaries from North America or the Far East. This paper represents the first study of floral anatomy and microstructure, as well as nectar composition, for a broad range of *Fritillaria* species, including, for the first time, taxa from two subgenera: *Korolkowia* Rix and *Liliorhiza* (Kellogg) Benth. & Hook.f. Our objectives were (1) to verify the presence of secretory tissues in selected members of the genus, (2) to investigate the microstructure of their nectaries, and (3) to compare nectar production and composition in the taxa studied in order to shed light on the evolution of their pollination systems.

## Materials and Methods

Plant material used for this study was obtained from *Fritillaria* species cultivated at the University of Warsaw Botanic Garden (BG), from the private collections of the coauthors (LH and PK) and from the private collection of Colin Everett (Somerton, Somerset, United Kingdom; CE). Many *Fritillaria* species are very rare in cultivation, and the number of specimens used for each type of analysis varied because of the availability of fresh plant material (accession numbers for species in this investigation are listed in **Table 1**).

**Table 1:** Investigated species of the subgenus *Fritillaria*.

	MIC	SEM	NEC	DIS	VOL	CON	NECTAR SUGAR PROFILE						SOU
							FRU	GLU	SUC	MAL	LAC	T	
*F. acmopetala*	+	+	24.2 ± 2.8 (86)	3.8 ± 0.6 (86)	34.8 ± 17.8 (42)	35.4 ± 15.7 (41)	76	23	1			132.2 (3)	>10 BG, LH, PK
*F. amana*	+	+	14.4 ± 2.2 (34)	4.7 ± 0.9 (29)	7.8 ± 2.3 (5)	47.8 ± 13.5 (5)	38	1	61			411.2 (1)	>10 BG, LH
*F. armena*	+	+	2.6 ± 0.9^∗^ (6)	0 (6)^∗^	42.6 ± 19.4 (4)	14.1 ± 11.2 (3)							6 BG
*F. aurea*	+	+	5.35 ± 0.5 (10)	6.5 ± 2.8 (10)		31 (1)							2 PK
*F. bithynica*	+		12.1 ± 0.8 (11)	1.2 ± 1.1^∗^ (6)			47	52	1			572.7 (1)	2 BG, PK
*F. carica*	+	+	2.6 ± 0.4 (36)	0.7 ± 0.1 (36)	13.6 ± 5.8 (14)	21.1 ± 12.7 (46)	47	44	7	2	?	344 (2)	>10 BG, PK
*F. caucasica*	+		10.5 ± 2.1 (18)	0.4 ± 0 (18)	1.4 ± 0.7 (3)	26.2 ± 11.9 (6)							5 BG,PK
*F. conica*	+		10.2 ± 11.6^∗^ (6)	1.8 ± 0.6^∗^ (6)		66.2 ± 3.4 (3)	30	30	40			310.82 (1)	3 BG
*F. crassifolia*	+	+	26.4 ± 1.1^∗^ (6)	2.7 ± 0.5^∗^ (6)			25	28	47			117.72 (1)	2 LH, PK
*F. davisii*	+	+	11.7 ± 3.4 (22)	3.2 ± 0.3^∗^ (17)									5 BG
*F. elwesii*	+	+	10.5 ± 1.7^∗^ (6)	0.8 ± 0.1^∗^ (6)	51 (1)	44 (1)	43	17	40			234.6 (2)	1 BG
*F. graeca*	+	+	5 ± 0.6 (6)	1.2 ± 0.2^∗^ (6)	9.6 ± 1 (11)	52.6 ± 10.7 (17)	45	40	15			217.4 (1)	>10 BG. PK. LH
*F. gussichiae*	+	+	23 ± 1.9 (6)	8.5 ± 0.5^∗^ (6)	44.5 ± 9 (4)^+^	14.5 ± 3.6 (4)							4 PK
*F. involucrata*	+	+	33.5 ± 4.3^∗^ (6)	4.3 ± 0.7^∗^ (6)		17 (1)	13		87			88.8 (1)	1 LH
*F. kotschyana*	+		13.9 ± 1.3 (24)	5.4 ± 0.8 (6)	55.3 ± 8.1 (4)	24.3 ± 7.5 (6)	73	27				133.9 (1)	>5 LH. PK
*F. latakiensis*	+	+	11.6 ± 1.1 (24)	1.2 ± 0.3 (24)		37.6 ± 7.1 (4)							4 LH
*F. lusitanica*	+		20.7 ± 5^∗^ (6)	3.3 ± 0.5^∗^ (6)	51^+^	9	27	3	70			156.5 (1)	1 LH
*F. meleagroides*	+	+	19.3 ± 3.5^∗^ (6)	4.7 ± 0.7^∗^ (6)	39.1 ± 7.7 (9)	31.6 ± 13.4 (11)	59	15	26			246.1 (1)	>10 LH. PK
*F. michailovskyi*	+	+	14.9 ± 5.1 (67)	3.6 ± 0.6 (34)	3.6 ± 3.5 (18)	17.4 ± 8.3 (8)	47	19	34			163.1 (3)	>10 BG
*F. minuta*	+	+	2.4 ± 0.5^∗^ (6)	1.4 ± 0 (6)	1.2 ± 1.4 (11)	64.9 ± 11.4 (13)							>10 BG. PK
*F. montana*	+		25.9 ± 2.7^∗^ (6)	4 ± 0.2^∗^ (6)	1.3 (1)	14.5 (1)							3 BG. PK.LH
*F. mutabilis*	+	+	12.2 ± 3.2^∗^ (6)	2.2 ± 0.5^∗^ (6)									3 LH
*F. obliqua*	+		2.5 ± 0.6^∗^ (6)	0.9 ± 0.2^∗^ (6)	2.8 (1)	61 (1)							1 PK
*F. olivieri*	+		12.5 ± 0.4 (24)	2 ± 0.4 (24)	11.6 ± 3.2 (4)	35.7 ± 8.1 (5)							>5 BG, LH, PK
*F. pallidiflora*	+	+	4.1 ± 1.0 (44)	5.4 ± 0.7 (35)	25.9 ± 18.2 (69)	43.7 ± 17.1 (70)	43	46	21			341.6 (1)	>10 BG
*F. pinardii*	+	+	2.3 ± 0.4^∗^ (6)	0.1 ± 0.0^∗^ (6)		40 (1)	49	47	1	3		282.2 (1)	3 BG. LH, PK
*F. pontica*	+	+	6.8 ± 0.8 (24)	3.2 ± 0.3 (24)	5.6 ± 5.8 (6)	16.05 ± 3.3 (6)	58	30	10			92.6 (2)	>5 BG
*F. pyrenaica*	+	+	15 ± 1^∗^ (6)	4.9 ± 0.7^∗^ (6)	52.3 ± 13 (6)	12.8 ± 4.5	37	14	49			200.4 (2)	>5 LH. PK
*F. ruthenica*	+		13.6 ± 0.9 (6)	2.4 ± 0.3^∗^ (6)	0.1 (1)								>5 BG. LH
*F. sibthorpiana*	+	+	3.8 ± 0.7^∗^ (6)	0.8 ± 0.2^∗^ (6)									1 BG
*F. stribrnyi*	+	+	4.7 ± 0.8^∗^ (6)	0.8 ± 0.0^∗^ (6)	7 (1)	35 (1)							1 BG
*F. thessala*	+	+	13 ± 2.3 (24)	5.10.3 (24)	14 (1)	16.2 ± 0.2 (3)	30	4	66			133.9 (2)	<5 BG. LH. PK
*F. thunbergii*	+		5.9 ± 0.9^∗^ (6)	1.1 ± 0.2^∗^ (6)									2 BG, PK
*F. tubiformis*	+	+	4.4 ± 0.3^∗^ (6)	5.7 ± 4.6^∗^ (6)									1 PK
*F. ussuriensis*	+		21 ± 3^∗^ (6)	1.6 ± 0.4^∗^ (6)	3.20 (1)	77.5 (1)	23	2	75			377.2 (1)	1 BG
*F. uva-vulpis*	+	+	11.9 ± 1.7 (32)	0.02 ± 0 (20)	12 ± 13.7 (25)	47.8 ± 17.1 (28)	37	41	22			241.4 (1)	BG. LH. PK
*F. verticillata*	+	+	4.8 ± 0.3 (4)	2.6^∗^ (4)	0.6^+^ (1)		48	52				781.8 (1)	1 LH
*F. whittallii*	+	+	14.6 ± 2.6 (28)	1.3 ± 0.1 (27)	13.5 ± 3.8 (4)	50.9 ± 7.3 (4)	46	13	41			405.8 (2)	5 BG. PK
**Subgenus *Japonica***											
*F. amabilis*	+	+	5.9 ± 0.3^∗^ (6)	0.8 ± 0.2^∗^ (6)			44	24	32			491.8 (1)	1 LH
*F. ayakoana*	+	+	5.9 ± 0.4^∗^ (6)	2 ± 0.4^∗^ (6)	0.9^+^ ± 0.7 (3)	40.7 ± 0.5 (3)							3 LH
**Subgenus *Korolkowia***											
*F. sewerzowii*	+	+	11.8 ± 3.9 (34)	0 (34)	24.6 ± 17.5 (33)	61.9 ± 11.7 (35)	50	50				280.6 (1)	>10 BG
**Subgenus *Liliorhiza***											
*F. affinis*	+		26.9 ± 5.5^∗^ (6)	4.7 ± 1^∗^ (6)	15.4	12	58	38	4			379.2 (1)	1 OB
*F. camschatcensis*	+	+	4.6 ± 0.7 (30)	0 (30)			58	40	2			41.3 (1)	>10 BG
*F. eastwoodiae*	+	+	2.5 ± 0.6^∗^ (6)	0.2 ± 0^∗^ (6)	33.8^+^ ± 7.2 (3)	16.3 ± 2.5 (3)	65	32	3			151.55 (1)	1 CE
*F. gentneri*	+	+	10.8 ± 3.4 (14)	2.2 ± 0.3 (14)	54 ± 9.8 (10)	31.1 ± 18.1 (8)	50	30	20			525.85 (2)	>5 BG,CE
*F. liliacea*	+	+	2.8 ± 0.6^∗^ (6)	0.4 ± 0.1^∗^ (6)	34^+^ (1)	48 (1)							1 CE
*F. recurva*	+	+	5. ± 0.2 (24)	1.3 ± 0.9 (24)	49.2 ± 18.3 (25)	33.1 ± 10.6 (25)	58	33	9			163.1 (1)	>5 BG. CE
**Subgenus *Petilium***											
*F. eduardii*	+	+	21.4 ± 1.4 (13)	2.6 ± 0.3 (13)	56.5 ± 18.3 (17)	5 ± 8.1 (24)	80	20				17.86	>10 BG, PK
*F. imperialis*	+	+	29.6 ± 1.8 (46)	1.5 ± 0.1 (46)	204.8 ± 94.7 (28)	13.6 ± 3.7 (20)	49	51	0			68	>10 BG
*F. raddeana*	+	+	2.8 ± 2.2 (38)	4.3 ± 0.5 (38)	8.7 ± 1.4 (8)	50.1 ± 15.7 (31)	43	30	52			227.7 (2)	>10 BG
**Subgenus *Rhinopetalum***											
*F. bucharica*	+		10.5 ± 0.7 (36)	0.8 ± 0 (36)	0.3 ± 0 (4)	52.7 ± 1.8 (5)							>10 BG
*F. gibbosa*	+	+	7.7 ± 0.8^∗^ (6)	0.1 ± 0^∗^ (6)									4 PK
*F. stenanthera*	+	+	11.6 ± 1.4 (30)	0.2 ± 0 (30)	0.6 ± 0.8 (25)	45.5 ± 15.9 (27)	32	63	5			218.7 (1)	>10 BG
**Subgenus *Theresia***											
*F. persica*	+	+	3.5 ± 0.4 (64)	2 ± 0.3 (54)	4.3 ± 4.5 (74)	46.5 ± 18.7 (69)						825.8 (1)	>10 BG
**Other species**											
*F. grandiflora*	+	+	14.7 ± 1^∗^ (6)	6.4 ± 0.6^∗^ (6)	42.3 ± 1.3 (2)	23.8 ± 2.8 (2)							2 PK
*F. olgae*	+		19.6 ± 1.9^∗^ (6)	2.5 ± 0.2^∗^ (6)	74.4 ± 33.2 (5)	29.5 ± 14 (5)	37	2	61			199.4 (1)	5 BG

*Investigated species of subgenera Japonica (F. amabilis and F. ayakoana), Korolkowia (F. sewerzowii), Liliorhiza (F. affinis, F. camschatcensis, F. eastwoodiae, F. gentneri, F. liliacea, F. recurva), Petilium (F. eduardii, F. imperialis, and F. raddeana), Rhinopetalum (F. bucharica, F. gibbosa, and F. stenanthera), Theresia (F. persica) and other species. MIC, microscopical analysis; staining with Lugol’s iodine solution and Sudan IV; SEM, scanning electron microscope; NEC, nectary size; DIS, distance from nectary to the base of the perianth, data presented as means (number of petals studied) ± SD referring to technical and biological replicates or only to technical replicates (marked with^∗^); VOL, nectar volume (when measured only from standing crop marked with ^+^); CON, nectar concentration, data presented as means (number of inflorescences studied) ± SD; Nectar sugar profile [%]: FRU, fructose; GLU, glucose; SUC, sucrose; MAL, maltose; LAC, lactose; T, total amount of sugars [mg/ml]; SOU, source and number of specimens obtained.*

### Microscopical Observations

All microscopical examinations were conducted for flowers in full anthesis. Flowers from 1 to 10 were selected for morphometric measurements of the nectary size and position. If less than three specimens were available, all flowers from one individual were measured, and if more plants were available, the flowers studied were selected randomly. The study was conducted with the use of a digital caliper Borletti DIN 862 (Borletti, Italy), tethered to a computer to record the values. Shape, size, structure, and color of the nectaries were observed in the fresh material using a Nikon SMZ 1000 stereomicroscope (Nikon Corp., Japan).

### SEM Observations

Three areas were selected for SEM observations on the outer tepals: the nectaries, the area distal to the nectaries, and the tip of the tepals. Sections of nectaries collected in the greenhouse and from PK collection were prepared by fixing nectary tissue in 2.5% glutaraldehyde in phosphate buffer (pH 7.4; 0.1 M). After three washes in phosphate buffer, they were postfixed in 2% (w/v) osmium tetroxide solution for 2 h and were dehydrated in a graded ethanol series. After dehydration, samples were subjected to critical point drying using liquid CO_2_ and were sputter-coated with gold. Nectaries gathered from the collections of LH and CE were transported from the United Kingdom to Warsaw in 70% ethanol. Subsequently, the material was prepared for SEM as described above, and the sample was examined using a SEM LEO 1430VP (Zeiss, Germany) and Zeiss Libra 120 (Zeiss, Germany).

Seven representative species (*F. eduardii*, *F. gentneri*, *F. michailovskyi*, *F. persica*, *F. recurva*, *F. raddeana*, and *F. sewerzowii*), either characterized by visually different nectary structures or representing closely related species, were prepared as semi-thin nectary sections.

Plant material was prepared by fixing nectary tissue in 2.5% glutaraldehyde in phosphate buffer (pH 7.4; 0.1 M). The samples were then washed three times before postfixation in 2% (w/v) osmium tetroxide solution for 2 h and were dehydrated in a graded ethanol series. After dehydration, they were infiltrated with LR White resin. Succeeding polymerization at 60°C, the nectaries sections were cut with a glass knife. The semi-thin sections (0.9–1.0 μm thick), stained with an aqueous solution of 1% methylene blue/1% Azure II (1:1) for 5–7 min on a hot plate (60°C), were prepared for LM and analyzed for general histology.

Hand-cut sections of the nectaries of all studied species were also prepared for histochemical investigations by means of LM. The size of epidermal and parenchymal cells and the depth of nectariferous tissue were measured. Subsequently, hand-cut sections were stained with an alcoholic solution of Sudan IV for lipids and with Lugol’s iodine solution for starch. Sections of nectaries were also stained with aniline blue and were examined by means of fluorescence microscopy (FM) in order to test for the presence of callose in cell walls.

### Nectar Sampling

Flowers for nectar sampling in the collections of BG and PK were first selected during the bud stage and were bagged with nylon mesh (net 0.5 mm) to prevent visits by insects. During anthesis but before anther dehiscence, nectar was sampled. In the BG collection flowers, progress was checked daily in the morning and in the afternoon for the presence of nectar. Nectar sampling in the LH and CE collections was from unbagged flowers open to animal visitors. All nectar was sampled with microcapillary pipettes from nectaries of all six tepals and was combined as one sample per flower. In the case of *F. camschatcensis*, nectar volumes were very small and sampling with microcapillaries pipettes was performed under a Nikon SMZ 1000 stereomicroscope (Nikon Corp., Japan). The collected nectar was subsequently expelled from microcapillaries onto a refractometer prism RL-4 (PZO, Poland) in order to measure nectar sugar concentration.

Nectar was also sampled to assess nectar sugar composition. Nectar from a standing crop of unbagged flowers was collected for this purpose. Particular care was taken during nectar collection, to avoid any contamination by pollen, phloem soap, or any other plant tissue. However, as most of the *Fritillaria* have downward facing flowers the risk of pollen contamination was low.

No attempts were made to emasculate flowers prior to sampling, so that sugar composition represents nectar as encountered by visitors. Nectar from one to three flowers of each species was placed into 1.5 ml Eppendorf tubes prior to analysis using high performance liquid chromatography (HPLC). The samples were frozen (−20°C) until required. Nectar was diluted with water to a volume of 50 μl (10 μl of nectar + 40 μl of water). The sample was filtered through spin columns using a 0.4 μm pore size membrane filter before injection. The supernatant was then loaded into the insert. An Agilent 1260 Infinity Series HPLC system with autoinjector, refrigerated autosampler compartment, thermostatted column compartment, quaternary pump with in-line vacuum degasser, and refractive index detector was used. A ZORBAX Carbohydrate Analysis Column (4.6 mm × 250 mm, 5 μm) was used for sugar separation and analysis. A 10 μl aliquot sample or standard solution was injected. The separation was conducted at 30°C with the mobile phase comprising acetonitrile:water (70:30, v/v) at a flow rate of 1.4 ml/min. The analytical data were integrated using the Agilent OpenLab CDS ChemStation software for liquid chromatography (LC) systems. Identification of sugars was performed by comparing retention times of individual sugars in the reference vs. test solution. The content of glucose, fructose, sucrose, maltose, and lactose was assayed based on comparisons of peak areas obtained for the samples investigated with those of the reference solutions.

## Results

### Nectary Location and Structure, Nectar Secretion, Concentration, and Composition

**FIGURE 1:**
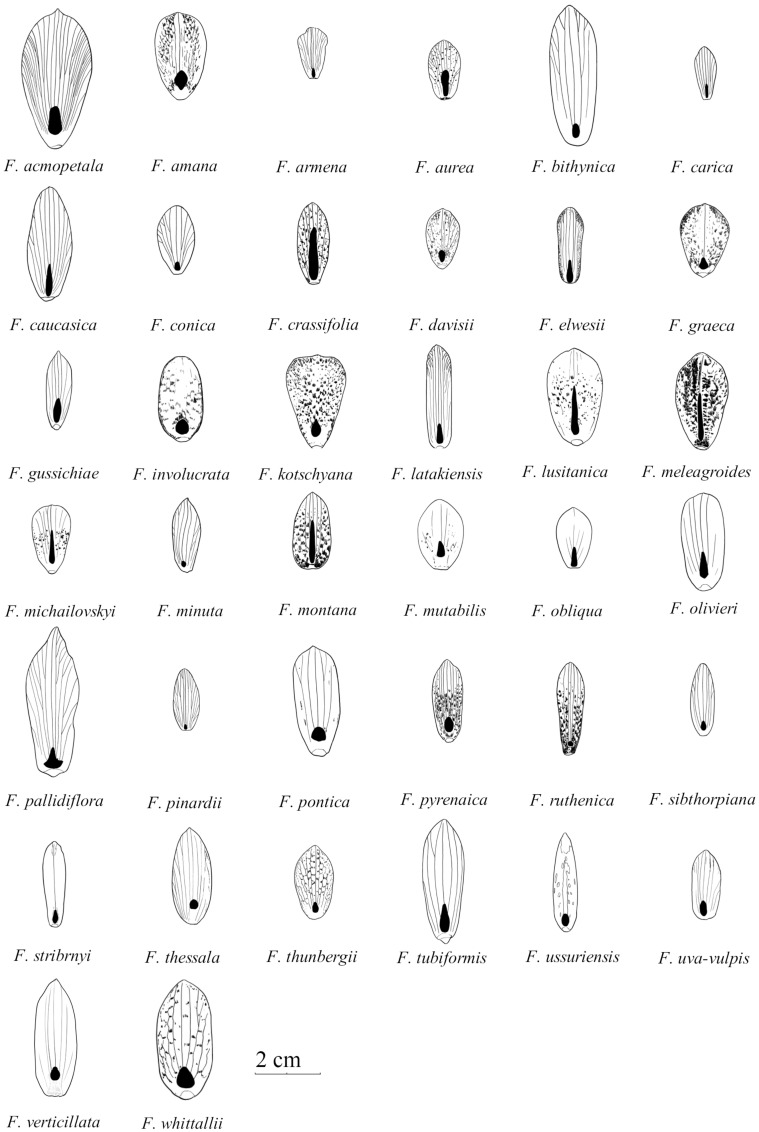
Schematic drawings of outer tepals and nectaries (filled with black) in species studied of the subgenus *Fritillaria*. Size graded according to natural size of studied tepals (Drawn by Jan Kryciński).

**FIGURE 2:**
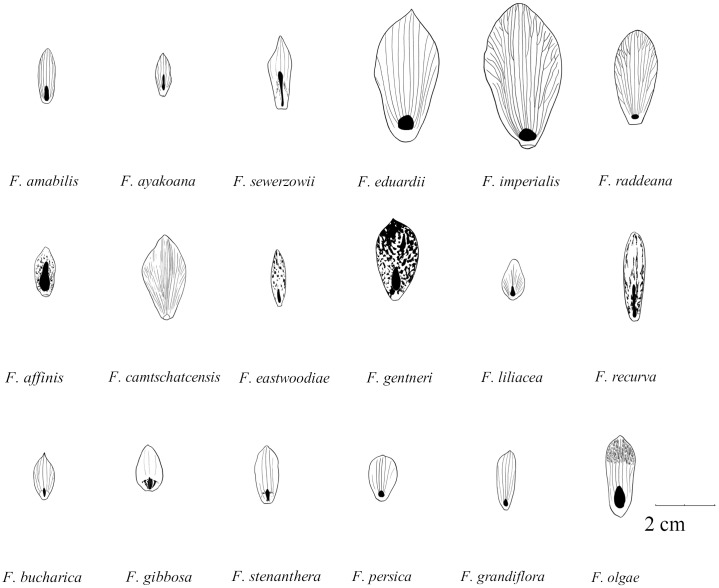
Schematic drawings of outer tepals and nectaries (filled with black) in selected species of subgenera: *Japonica* (*F. amabilis and F. ayakoana*)*; Petilium* (*F. eduardii, F. imperialis, and F. raddeana*); *Liliorhiza* (*F. affinis, F. camschatcensis, F. eastwoodiae, F. gentneri, F. liliacea, and F. recurva*)*; Rhinopetalum* (*F. bucharica, F. gibbosa, and F. stenanthera*)*; Korolkowia* (*F. sewerzowii*)*; Theresia* (*F. persica*); and other species (*F. olgae and F. tubiformis*) (Drown by Jan Kryciński).

In all species, six nectaries were located at the base of the tepals (**Figures 1**, **2**). The mean distance from the base of the perianth for all species studied was 2.3 ± 2.1 mm (means calculated only for technical replicates, if only one specimen was available, or means resulting from technical replicates were used to represent each biological replicate; missing SD values represent a single accession), in the range of 0.0 to 8.5 mm (**Table 1**). In all but one studied species, the nectaries of both outer and inner tepals were equally accessible to potential pollinators. Only in *F. persica* were the nectaries of the outer tepals not visible.

**FIGURE 3:**
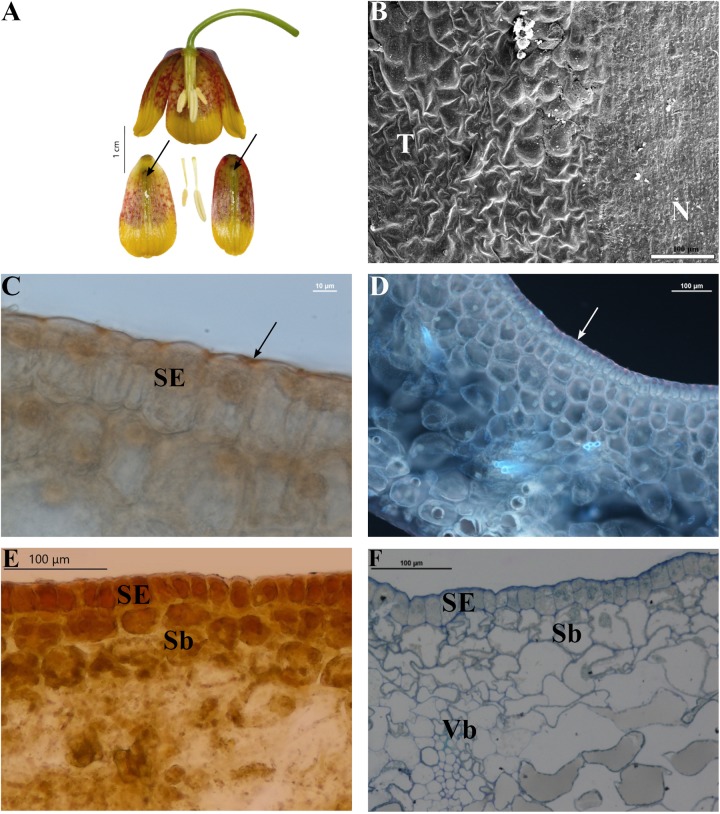
Flowers and nectaries of *F. michailovskyi* at full anthesis. Macro, SEM, and LM images. **(A)** Flowers and tepals, nectaries marked with arrows. **(B)** Part of outer tepal showing flat nectary cells (N) and slightly convex cells of surrounding tepals area (T). **(C)** Cuticule on the surface of secretory epidermis (SE) of outer tepals stained with Sudan IV, arrow indicates lipids layer on the surface of cuticule of nectary. **(D)** Staining with aniline blue does not reveal the presence of callose in the walls of nectary cells. Arrow indicates lipids on the cuticle of the nectary of outer tepals. **(E)** Treatment with Lugol’s iodine solution reveals the absence of starch in secretory (SE) and subsecretory parenchyma (Sb). Secretory epidermis with dense cytoplasm. **(F)** Staining with Azure II showing secretory epidermis (SE), subsecretory parenchyma (Sb), and ground parenchyma with vascular bundle (Vb).

**FIGURE 4:**
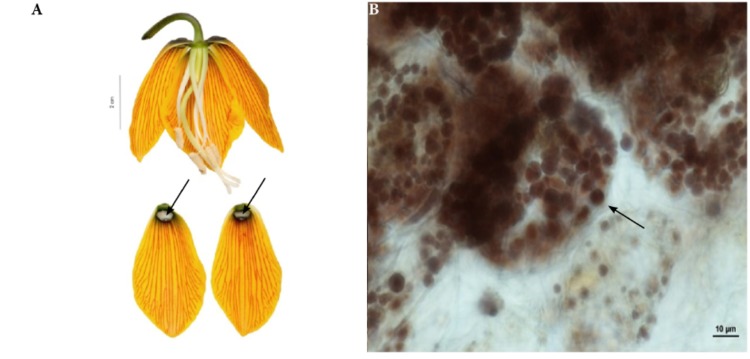
Flowers and nectaries of *F. imperialis* at full anthesis. Macro and LM images. **(A)** Flowers and tepals, nectaries marked with arrows. **(B)** Treatment with Lugol’s iodine solution reveals the presence of starch in secretory and subsecretory parenchyma. Grains visible in the plastids of subepidermal cells in *F. imperialis* outer tepal nectaries.

Nectary cells were smaller, flatter, and more regular in shape than other epidermal cells (not shown). In each case, the nectaries consisted of a single-layered epidermis (without stomata) and several layers of subepidermal parenchyma (**Figures 3D,E**)^1^. The cytoplasm of epidermal cells contained a large nucleus, small vacuoles, and plastids. Plastids were also present in deeper layers of the nectaries’ parenchyma. Vascular bundles contained both xylem and phloem elements. Subepidermal nectary parenchyma consisted of 2–5 layers (**Figure 3F**). Staining with Lugol’s iodine solution revealed no starch grains, with the exception of members of the subgenus *Petilium*, where staining revealed the presence of numerous starch grains in the plastids of epidermal and subepidermal cells (**Figure 4B**).

Staining with Sudan IV revealed the presence of numerous droplets of lipid on the epidermis (**Figure 3C**) and within nectary cells of all studied species, the cuticule on the surface of secretory epidermis stained red. Staining with aniline blue did not reveal the presence of callose in cell walls (**Figure 3D**). The mean area occupied by the nectaries of all studied species was 11.8 ± 8.6 mm^2^, in the range of 1 to 38.2 mm^2^ (**Table 1**).

In all of the investigated species, each of the six nectaries located adaxially on perianth segments produced nectar. Nectar passed across the cell wall and was exuded through pores in the cuticle.

The amount of nectar produced depended largely based on the species. On average, *Fritillaria* flowers produced 30.6 ± 52.2 μl of nectar, in the range of 0.4 to 204.8 μl (means and SDs; *N* = 498, 41 species; data pooled for all seasons and species investigated). The concentration of nectar, on average, was 39.1 ± 20.63%, in the range of 5 to 77.5% (*N* = 599, 44 species). Nectar in most species was hexose-rich (with mean total concentration of 278.6 ± 187.8 mg/ml). The sugar profile of nectar was dominated by sucrose and glucose, which were also detected in the nectar of all species (115.4 ± 77.2 mg/ml and 88.5 ± 122.2 mg/ml, respectively; *N* = 54, 34 species). Fructose was also a significant component of *Fritillaria* nectar (87.6 ± 84.4 mg/ml), but it was not present in the nectar of all species studied. Traces of maltose and lactose were also detected in the nectar of several species (4 ± 1.8 and 5.1 mg/ml, respectively) (**Table 1** and **Supplementary Material**).

### Subgenus *Fritillaria*

**FIGURE 5:**
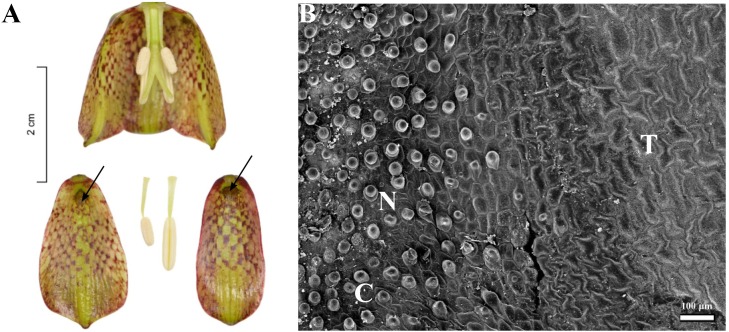
Flowers and nectaries of *F. pyrenaica*. Macro and SEM images. **(A)** Flowers and tepals, nectaries marked with arrows. **(B)** Part of outer tepal showing the nectary area (N), comprising conical papillae and slightly convex cells (C) of surrounding tepals area (T).

This subgenus was represented by 38 species (**Table 1**). The nectaries of this subgenus were highly variable, and the nectaries differed greatly in size and area occupied (mean value 12.4 ± 8.8 mm^2^). The average distance of the nectaries from the base of the perianth was 2.7 ± 2.2 mm. Several species had ovate nectaries of uniform background color (i.e., of poor contrast) and were difficult to differentiate. For example, *F. amana* had round nectaries, sometimes encircled by a brownish band, especially those of the inner tepals, but the nectaries were generally of a uniform green color. *Fritillaria* *aurea*, *F. bithynica*, *F. conica*, and *F. sibthorpiana* had round, slightly depressed, yellow or slightly greenish nectaries, similar in color to the rest of the tepal. *Fritillaria* *davisii* and *F. pyrenaica* (**Figure 5A**) had linear-lanceolate darker nectaries that did not contrast well against the tessellated brownish background. *Fritillaria* *elwesii* had ovate to triangular, greenish nectaries, of a slightly darker hue when compared to that of surrounding tissues. *Fritillaria* *pallidiflora* had triangular nectaries, greenish to yellowish and of the same color as the tepals.

*Fritillaria* *uva-vulpis* had ovate yellowish nectaries and a similarly colored background.

Considerably, more members of the subgenus *Fritillaria* had contrasting nectaries. *Fritillaria* *acmopetala*, *F. graeca*, *F. involucrata*, *F. latakiensis*, *F. mutabilis*, *F. olivieri*, *F. pontica*, *F. thessala*, and *F. verticillata* had ovate to obovate, dark nectaries that contrasted strongly against the brighter tepals. *Fritillaria* *kotschyana* and *F. whittallii* also had ovate to obovate, dark nectaries that contrasted strongly against the tessellated tepals. *Fritillaria* *minuta* had ovate, slightly depressed nectaries of dark green color and contrasting yellowish tepals. Similarly, *F. pinardii* and *F. carica*, had linear-lanceolate, deeply depressed, greenish nectaries, that were only slightly darker than the green-yellow tepals. Sometimes, there was slightly more contrast when the tepals were yellow. *Fritillaria* *lusitanica*, *F. ussuriensis*, *F. ruthenica*, and *F. michailovskyi* (**Figure 3A**) had greenish, oblanceolate nectaries surrounded by reddish, tessellated tepals. *Fritillaria* *thunbergii* had greenish, oblanceolate nectaries surrounded by yellowish, tessellated tepals. *Fritillaria caucasica*, *F. obliqua*, and *F stribrnyi* had linear-lanceolate, bright green nectaries contrasting with dark red tepals, like those of *F. montana*, where the green, slightly depressed nectaries were surrounded by red, tessellated tepals. *Fritillaria* *gussichiae* had ovate, bright green nectaries that contrasted strongly against a dark red background. *Fritillaria* *crassifolia* had linear nectaries that were usually green and heavily marked with purple. Nectaries were visible but did not contrast strongly against the green, red-tessellated tepals. Similarly, in *F. meleagroides*, the dark, linear nectaries were surrounded by green tepals with dark red tessellation. *Fritillaria* *armena* had nectaries at the base of the perianth. Several species had nectaries close to the base of the perianth (not more than 1 mm distant) or more than 5 mm from it, but for most of the species nectaries arose more than 1 mm but less than 5 mm from the base of the perianth (**Table 1**).

Scanning electron microscope analysis revealed that in most of the species investigated, the internal surface of the nectary was flat, while the surrounding area and the rest of the tepal was slightly undulate, owing to the slightly convex cells (**Figure 3B**). Nectaries of *F. verticillata* also had slightly convex cells. In *F. armena*, the area of the nectary was also surrounded by a row of elevated, rounded protrusions, also present on the tepals, where they were arranged in rows. In *F. davisii*, the remainder of the tepal was covered with elevated protrusions. *Fritillaria* *uva-vulpis* and *F. michailovskyi* had rows of elevated cells directly above the nectaries. In *F. pyrenaica* (**Figure 5B**), the area of the nectary was comprised of conical papillae. In *F. tubiformis*, nectary cells had papillae and the epidermal cells of the surrounding area were also slightly convex.

Plants of this subgenus produced variable amounts of nectar (32.3 ± 54.4 μl) of highly variable concentration (38.5 ± 20.6%.) The lowest mean concentration was recorded for *F. pyrenaica* (12.8 ± 4.5%, 52.3 ± 13 μl). The highest mean value was observed for *F. ussuriensis* (77.5%, 3.2 μl). The highest production was recorded for *F. kotschyana* (55.3 ± 8.1 μl, 24.3 ± 7.5%). The smallest volume was recorded for *F. verticillata* (0.6 μl, but this was too small to measure sugar concentration) (**Table 1**).

### Subgenus *Japonica*

**FIGURE 6:**
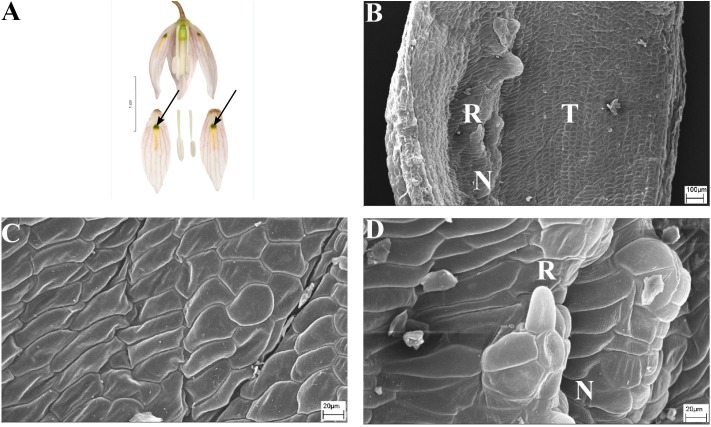
Flowers and nectaries of *F. ayakoana*. Macro and SEM images. **(A)** Flowers and tepals, nectaries marked with arrows. **(B)** Part of outer tepal showing the nectar-bearing area (N) with upwardly curved ridge or protuberance on its surface and slightly convex surrounding area (T). **(C)** Slightly convex cells of outer tepal surface directly above the nectary. **(D)** Cuticule of the nectary (N) of outer tepal with protuberance (R).

This subgenus was represented by two species (**Table 1**). Nectaries of these species were yellowish and ovate-lanceolate. In the case of *F. ayakoana* (**Figure 6A**), the base of the nectary was green and it contrasted strongly with the bright tepals. In the middle of the nectary, there were small upwardly curved ridges or protuberances. The area occupied by nectaries of the members of this subgenus varied little and, on average, measured 5.4 ± 1 mm^2^. The nectaries were placed close to the base of the perianth (**Table 1**).

Scanning electron microscope analysis revealed that the surface of the nectaries and the surrounding areas of *F. ayakoana* (**Figures 6B,C**) flowers were identical with conical projections (**Figures 6B,D**).

The layer of subepidermal nectary parenchyma was deeper in *F. ayakoana* and was four or more cells deep, whereas in *F. amabilis*, it was 2–3 cells deep.

During anthesis, the entire nectary area was coated with nectar (no data regarding nectar replenishing available). Flowers of the species studied produced, on average, 0.9 ± 0.7 μl nectar of concentration 40.7 ± 0.5% (**Table 1**).

### Subgenus *Korolkowia*

**FIGURE 7:**
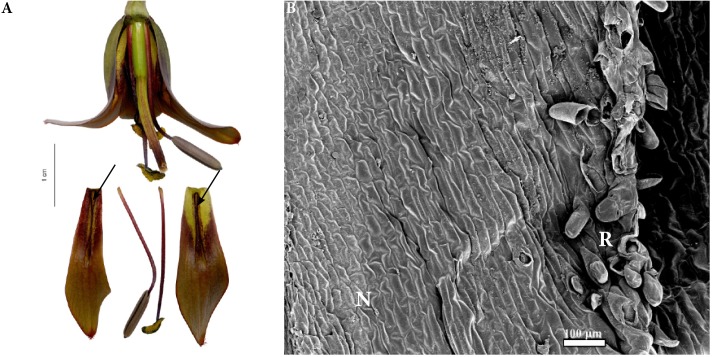
Flowers and nectaries of *F. sewerzowii*. Macro and SEM images. **(A)** Flowers and tepals, nectaries marked with arrows. **(B)** Part of outer tepal showing the nectary (N), surrounded by papillose ridges (R).

This is a monotypic subgenus containing *F. sewerzowii*. Its nectaries were long, elliptical, and depressed in a groove (**Figure 7A**), which was surrounded by a row of longitudinal papillose ridges (**Figure 7B**). They did not cover the nectaries, which were clearly visible, green, and were strongly contrasting with the dark background. The area of the nectaries was measured to be 11.8 ± 3.9 mm^2^, and the nectaries were located at the base of the perianth (**Table 1**).

Scanning electron microscope analysis revealed that the surface of the nectaries was slightly undulate and wrinkled. The cells of the surrounding area were slightly convex (**Figure 7B**).

During anthesis, the entire nectary was coated with nectar, which was replenished on its removal. Flowers of this species produced, on average, 24.6 ± 17.5 μl nectar of concentration 61.9 ± 11.7% (**Table 1**).

### Subgenus *Liliorhiza*

**FIGURE 8:**
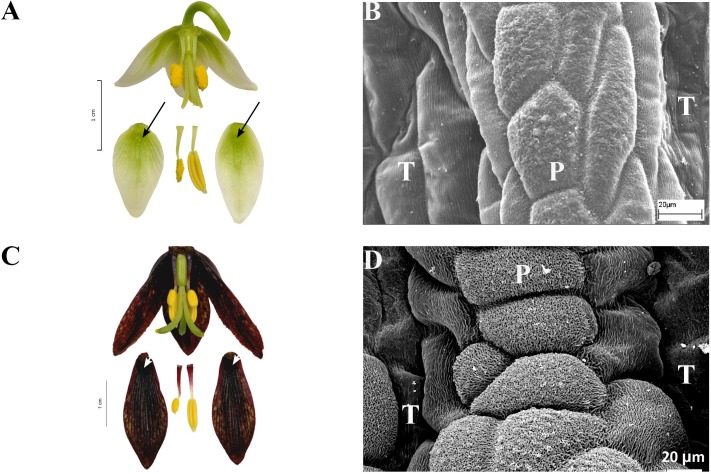
Flowers and nectaries of *F. liliacea* **(A,B)** and of *F. camschatcensis* **(C,D)**, both in full anthesis. **(A)** Flowers and tepals of *F. liliacea*, nectaries marked with arrows. **(B)** Protrusions surrounding the nectary area (P) on *F. liliacea* outer tepal (T). **(C)** Flowers and tepals of *F. camtschatcensis*, nectaries marked with arrows. **(D)** Protrusions surrounding the nectary area on the outer tepal of *F. camschatcensis*.

**FIGURE 9:**
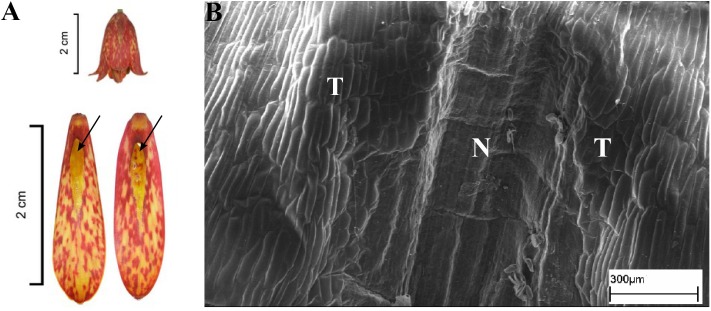
Flowers and nectaries of *F. gentneri*. Macro and SEM images. **(A)** Flowers and tepals, nectaries marked with arrows. **(B)** Part of outer tepal showing deeply depressed nectary (N).

This subgenus was represented by six species (**Table 1**). Nectaries of *F. camschatcensis* were very narrow, lanceolate and were hidden in the ridges (**Figure 8C**). Its surface was covered with proturbance and it glistened; therefore, the nectaries always looked as if they contained nectar. The nectaries of other *Liliorhiza* species were ovate-lanceolate in shape and were not protected by any additional structures. Nectaries of *F. affinis*, *F. recurva*, *F. gentneri*, and *F. eastwoodiae* were similar in appearance and were brightly colored against a contrasting darker, tessellated background (**Figure 9A**). The nectaries of *F. liliacea* (**Figure 8A**), like the surrounding part of the tepal, were uniformly green and, thus, almost invisible. *Fritillaria affinis* had the largest nectaries (29.6 ± 5.5 mm^2^), and the smallest nectaries were recorded for *F. liliacea* (2.8 ± 0.6 mm^2^). The nectaries were generally situated close to the base of the perianth (0.9 ± 0.9 mm) (**Figure 9A** and **Table 1**).

Scanning electron microscope analysis revealed that the surface of the depressed nectaries of *F. eastwoodiae* consisted of slightly convex epidermal cells, as well as in the surrounding area. *Fritillaria liliacea* and *F. camschatcensis* also had depressed nectaries surrounded by a row of elevated cells having a grooved surface (**Figures 8B,D**). Both *F.* *gentneri* and *F. recurva* had depressed nectaries surrounded by elevated cells (**Figure 9B**).

The subepidermal nectary parenchyma consisted of four or more layers. Only in *F. eastwoodiae* was the nectary parenchyma shallower and consisted of 2–3 layers.

In *F. gentneri* and *F. recurva*, nectar was replenished on its removal. *Fritillaria* *camschatcensis* produced very small amount of barely visible, viscous nectar. Owing to the consistency of the nectar and the fact that the nectary surface was glistening, it was not possible to assess nectar replenishment. No data was available for *F. eastwoodiae* and *F. liliacea*. Plants of this subgenus produced copious amounts of nectar (48 ± 17.1 μl) of average concentration 30.1 ± 11.9%. The lowest concentration of nectar was recorded for flowers of *F. affinis* (12%), and the highest was recorded for *F. liliacea* (48%). The smallest volume of nectar was produced by *F. affinis* (15.4 μl), and the greatest volume of nectar was produced by *F. gentneri* (54 ± 9.8 μl) (**Table 1**).

### Subgenus *Petilium*

**FIGURE 10:**
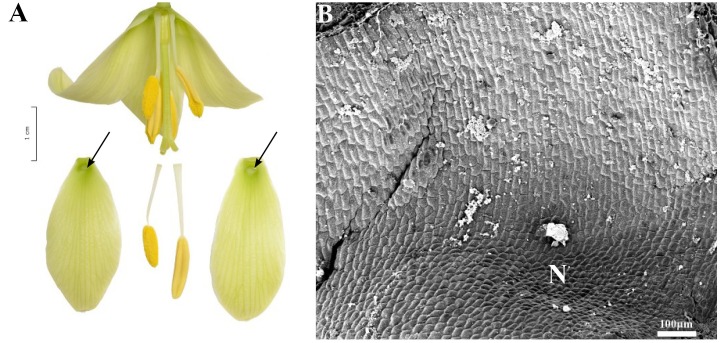
Flowers and nectaries of *F. raddeana*. Macro and SEM images. **(A)** Flowers and tepals, nectaries marked with arrows. **(B)** Part of outer tepal showing depressed, cup-shaped nectary (N), and slightly convex cells of surrounding area.

This subgenus was represented by three species (**Table 1**). The nectaries were depressed, elliptic, or round in shape. In *F. imperialis* and *F. eduardii*, they were similar in size with an area of 27.7 ± 4.7 mm^2^ and were similarly located 1.7 ± 0.7 mm above the base of the perianth. Nectaries of *F. raddeana* (**Figure 10A**) were smaller (2.8 ± 2.2 mm^2^) and were located 4.3 ± 0.5 mm above the base of the tepal (**Table 1**). The white nectaries of *F. imperialis* (**Figure 4A**) and *F. eduardii* contrasted sharply with the surrounding dark green background. Nectaries of *F. raddeana* were not as strongly contrasting as were those of the two previous species described; they were darker and surrounded by a similarly dark background.

Scanning electron microscope analysis revealed that the inner surface of nectaries of *F. imperialis* and *F. eduardii* were flat, whereas the surrounding area and the rest of the tepals were slightly undulate. In *F. raddeana*, the area within the nectary was similar to the remainder of the tepal area and was also slightly undulate (**Figure 10B**). Subepidermal nectary parenchyma consisted of 2–4 layers. Staining with Lugol’s iodine solution revealed the presence of numerous starch grains in the plastids of epidermal and subepidermal cells (**Figure 4B**).

During anthesis, the entire nectary area was coated with nectar. It was easily accessible in the form of large droplets. Nectar was replenished on its removal. Flowers of this subgenus produced, on average, 133.3 ± 107.5 μl of nectar of concentration 26.2 ± 23.1%. The highest concentration and the smallest volume were recorded for *F. raddeana* (50.1 ± 15.7% and 8.7 ± 1.4 μl, respectively). The lowest concentration was recorded for *F. eduardii* (5 ± 8.1%). Flowers of *F. imperialis* produced the largest volume of nectar recorded for the subgenus *Petilium* (204.8 ± 94.7 μl) (**Table 1**).

### Subgenus *Rhinopetalum*

**FIGURE 11:**
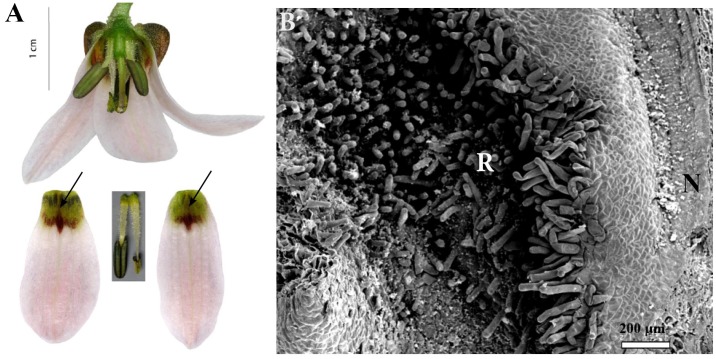
Flowers and nectaries of *F. stenanthera*. Macro and SEM images. **(A)** Flowers and tepals, nectaries marked with arrows. **(B)** Part of outer tepal showing deeply depressed spurs nectary (N), protected by densely papillose ridges (R).

This subgenus was represented by three species (**Table 1**). The typical nectaries were deeply depressed spurs, bearing two densely papillose ridges (**Figure 11A**). The ridges were adpressed, protecting access to the nectaries. In *F. stenanthera*, there were two additional papillose ridges adjacent to the nectary area (**Figure 11B**). Nectaries were visible on the reverse side of the tepals as dark “horns.” These projections differed from species to species; in *F. gibbosa*, one of the tepal “horns” was always significantly larger than the other. In *F. stenanthera*, all projections were of the same size. In *F. bucharica*, they were of similar size – more prominent at the bud stage, becoming flatter in mature flowers. In this species, nectaries were uniformly green, like the background, but the nectaries at the top were darkly spotted, located just above the entrance to the nectary. In *F. gibbosa*, the area surrounding the nectaries was dark brown, but the ridges were paler. Nectaries of *F. stenanthera* had both green and brown elements (**Figure 11A**). The surrounding area was mostly brown, and the sides were greenish or dark yellow. The deeply depressed area within the spur of the nectaries of *Rhinopetalum* was densely clothed with short cilia. Nectaries were flat and glabrous (**Figure 11B**).

It was difficult to measure the size of nectaries for representatives of *Rhinopetalum*, since, they were hidden inside the spur, and the borders of the nectary were not obvious. Moreover, in *F. gibbosa*, one of the nectaries was significantly larger. The approximate mean size of the nectary for members of this genus was 7.9 ± 3.6 mm^2^. The largest nectaries were recorded for *F. stenanthera* (11.6 ± 1.4 mm^2^), and the smallest nectaries were recorded for *F. gibbosa* (7.7 ± 0.8 mm^2^). Nectaries of all species were located very close to the base of the perianth, the mean value of the distance to the perianth being 0.6 ± 0.3 mm (**Table 1**).

Scanning electron microscope analysis revealed that the surface consisted of three distinct parts. The area of nectar secretion was represented by a depressed groove having a flat surface. It was surrounded by slightly elevated walls and had a slightly undulate surface. The rest of the spur was densely coated with short cilia. The remainder of the tepal was slightly undulated (**Figure 11B**).

Subepidermal nectary parenchyma consisted of four or more layers, and the nectar-bearing area occupied a relatively narrow region located at the center of the tepals.

During anthesis, the entire nectary area was coated with nectar, although it was not visible and was protected by the papillose ridges. Nectar was replenished on its removal. Flowers of this subgenus produced, on average, 0.5 ± 0.8 μl of nectar of concentration 46.6 ± 14.8%. On average, *F. bucharica* produced 0.3 ± 0 μl of nectar of concentration 52.7 ± 1.8%, with *F. stenanthera* producing 0.6 ± 0.8 μl and having the lower concentration of 45.5 ± 15.9% (**Table 1**).

### Subgenus *Theresia*

**FIGURE 12:**
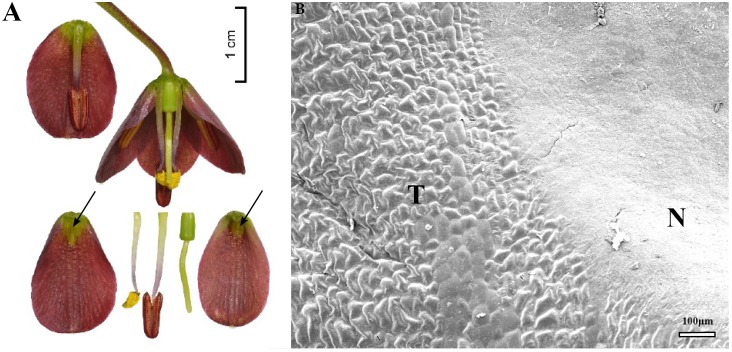
Flowers and nectaries of *F. persica*. Macro and SEM images. **(A)** Flowers and tepals, nectaries marked with arrows. **(B)** Part of outer tepal showing the flat nectary area (N) and slightly convex cells of surrounding area (T).

The nectaries of *F. persica* were slightly depressed and triangular in shape. They occupied an area measuring 3.5 ± 0.4 mm^2^ and were located 2 ± 0.3 mm above the base of the perianth (**Figure 12A** and **Table 1**). The green nectaries contrasted sharply with the surrounding dark purple background. However, several nectary cells were pigmented.

Scanning electron microscope analysis revealed that the inner surface of nectaries was flat, whereas the surrounding area and the remainder of the tepal bore slightly convex cells (**Figure 12B**). Subepidermal nectary parenchyma consisted of 3–5 layers.

During anthesis, the entire nectary area was coated with nectar, which extended beyond the nectary. Nectar from the inner tepals was easily accessible and lacked projections for protection, but nectar from the outer tepals was concealed behind the inner tepals. Nectar was replenished on its removal.

*Fritillaria* persica produced, on average, 4.3 ± 4.5 μl of nectar of concentration of 46.5 ± 18.7% (**Table 1**).

### Other Species

We also studied the nectaries of *F. grandiflora* and *F. olgae*, which are not classified into any subgenus (Rix, 2001). However, both of them presumably belong to the subgenus *Fritillaria* (Rix, 1974; Kiani et al., 2017). *Fritillaria* *grandiflora* had darkly colored, round nectaries, surrounded by tessellated tepals. *Fritillaria* *olgae* had ovate to triangular, darkly colored nectaries that contrasted with the green tepals. Nectaries of *F. grandiflora* measured 14.7 ± 1 mm^2^ and were located 6.4 ± 0.6 mm from the base of the perianth, for F. olgae, these values were 19.6 ± 1.9 mm^2^ and 2.5 ± 0.2 mm, respectively (**Table 1**).

Scanning electron microscope analysis revealed that the inner surface of the nectary was flat, whereas the cells of the surrounding area and the remainder of the tepal were slightly convex. Subepidermal nectary parenchyma consisted of 3–5 layers.

During anthesis, the entire nectary area was coated with nectar. Unless collected, large droplets of nectar were found at the edges of tepals in *F. olgae*. Nectar was replenished on its removal. *Fritillaria* *grandiflora* produced, on average, 42.3 ± 1.3 μl nectar of concentration 23.8 ± 2.8%. *Fritillaria* *olgae* produced, on average, 74.4 ± 33.2 μl of concentration 29.5 ± 14% (**Table 1**).

## Discussion

The present study reports SEM and LM analyses and descriptions of the nectaries for 56 species of *Fritillaria* contained in seven subgenera, including 29 species which were studied for the first time. This study is also the first to examine nectary surface of members of subgenera *Japonica*, *Korolkowia*, *Liliorhiza*, and *Theresia*. The most likely area used as a landing site for insect pollinators was imaged under SEM.

We also conducted SEM studies of the relevant area, which might be considered as the probable landing site for insect pollinators.

The nectaries of *Fritillaria* are positioned adaxially on each of the six perianth segments (**Figures 1**, **2**). However, several other nectary features, such as size, shape, or color, were generally variable among species. Such variation in shape and position of the nectaries was previously described by Rix and Rast (1975), Bakhshi Khaniki and Persson (1997), and Kiani et al. (2017), who studied the nectaries of 31 taxa from four subgenera using SEM and LM.

Despite differences in the appearance of nectaries, their morphology and positon were similar within the different subgenera. However, our studies echoed the conclusions of Bakhshi Khaniki and Persson (1997) that morphology and position of nectaries might be important diagnostic features in the taxonomy of the genus, as the nectaries of different subgenera vary greatly. In contrast, nectary ultrastructure, which is similar among all the species studied, does not provide any taxonomic information. The only exception is the subgenus *Petilium*, which is distinguished from other subgenera by the presence of starch in nectary cells.

We examined the nectaries for the occurrence of callose, which may push the projections into the cytoplasm and facilitate deposition of wall material (Offler et al., 2003), but we did not observe it in the cell walls of any species, although it was previously detected as the component of wall ingrowths in *F. meleagris* by Stpiczyńska et al. (2012). This difference might be species-specific (*F. meleagris* was not included in this study) or dependent on the flowers’ development stage. Clearly, this needs further studies. However, the outer epidermal cells and/or nectary cells in all species contained lipid droplets. The presence of a lipid layer on the plant surface may provide a way to reduce water loss. However, the role of lipid within nectary cells is in need of investigation (Kamińska and Stpiczyńska, 2011).

The present study also provides information about nectar sugar composition for 34 species and the quality and volume of this reward for additional 46 species. Since the nectar properties, like concentration and amount available for floral visitors, are highly variable, it cannot serve as a taxonomic tool. Also, contrary to Rix and Rast (1975), our study indicates that the fructose/glucose ratio varies greatly within and between the species studied; therefore, it does not provide useful taxonomic information.

### Subgenus *Fritillaria*

Usually, nectaries are more or less flat, surrounded by an area with slightly convex cells. Only in *F. pyrenaica* did we find the area around the nectary to be comprised of dense conical papillae, a feature that had not been previously described for any other *Fritillaria* species. In several species like *F. davisii*, *F. uva-vulpis*, and *F. michailovskyi*, there are ‘warts’ on the tepals or on the border of the nectary (Bakhshi Khaniki and Persson, 1997).

Rix and Rast (1975) studied nectar properties for several members of the subgenus *Fritillaria*. We obtained similar results for *F. bithynica*, *F. elwesii*, and *F. pyrenaica*. In comparison with the other species studied by Rix and Rast (1975), *F. pallidiflora, F. crassifolia, F. michailovskyi, F. acmopetala*, and *F. pontica* had glucose-dominant nectar in this study. *Fritillaria* *amana* produced sucrose-dominant nectar (H:S in the ratio of 2:3). Differences in the results obtained might be related to the method used (gas liquid chromatography vs. HPLC). However, it is evident that many complex factors affect nectar properties such as time of collection, weather conditions while sampling, or variation in the nectar properties depending on the development of the inflorescence (Willmer, 2011).

### Subgenus *Japonica*

Scanning electron microscope analyses and studies of nectar properties were conducted for the first time for species in the subgenus *Japonica*. The structure of the nectary and tepal surface was similar to that of other *Fritillaria* species studied. However, in the middle of the nectar-bearing area of *F. ayakoana* (**Figures 6B,D**) was a small, upwardly curved ridge or protuberance, a feature previously described for *F. kaiensis* and *F.* *japonica* (Naruhashi et al., 1997). Similar ridges also occur in the closely related subgenus *Rhinopetalum* (Bakhshi Khaniki and Persson, 1997). We conducted the first nectar sugar analysis for *F. amabilis* (the volume of *F. ayakoana* nectar was too small to be collected in the field). It was similar to that found in other fritillary species investigated, that is, hexose-dominant, with a relatively high sugar concentration (41%). However, the amount of nectar produced was considerably lower than the mean value for the genus, and again it was similar to the values detected in members of the closely related subgenus *Rhinopetalum*.

### Subgenus *Korolkowia*

Scanning electron microscope analyses and studies of nectar properties were conducted for the first time for *F. sewerzowii*. The tips of *F. sewerzowii* nectaries are visible from outside the flower; however, the main part is concealed within the very narrow, bell-shaped perianth (**Figure 7A**).

The surrounding papillose ridges only partly restrict access to the nectaries, in contrast to the ridges present in the members of *Rhinopetalum*, which almost completely cover the nectary. In this study, nectary area and floral reward were studied for the first time. The nectary area, unlike that of most fritillaries, is not flat, but it is slightly undulate and wrinkled (**Figure 7B**). Surprisingly, flowers of this species produced nectar without any trace of sucrose, a result which was previously only described for *F. imperialis* (Rix and Rast, 1975).

### Subgenus *Liliorhiza*

Nectaries and nectar in this subgenus were studied for the first time. SEM analysis revealed that the nectaries of *F. liliacea* (**Figure 8B**) were surrounded by a row of elevated cells with grooved surfaces, similar to those found in *F. camschatcensis* (**Figure 8D**). Flowers of most members of this subgenus produced copious nectar (mean value 48–49 μl) of medium sugar concentration (mean value 31%); only in the flowers of *F. camschatcensis* were traces of viscous nectar found. Nectar was hexose-dominant, although, the nectar of *F. gentneri* also contained a substantial amount of sucrose (20%).

### Subgenus *Petilium*

In this study, the nectaries of the subgenus *Petilium* differed from the other species studied. They are elliptical (Bakhshi Khaniki and Persson, 1997) and depressed. Anatomical studies revealed large accumulations of starch (**Figure 4B**), which was not previously reported for *Fritillaria* nectaries. Large amounts of starch, found, for example, in bee-pollinated *Anemopaegma album* (Bignoniaceae), are thought to be responsible for the secretion of large amounts of sugar during the peak secretory period (Dafni and Vereecken, 2016; Guimaraes et al., 2016). Furthermore, the white, glistening appearance of nectaries within the subgenus *Petilium* may also result from the presence of starch; since, the flat upper epidermis may act as a thin film reflector responsible for its glossiness. It may further serve as a filter to backscattered light as the starch bodies located in the parenchyma layers have strong light-scattering properties, as described for *Ranunculus* spp. (van der Kooi et al., 2017). Moreover, the convex-shaped nectaries always appear full of nectar, even when they are empty.

An earlier study (Rix and Rast, 1975) considered *F. imperialis* to be distinct within *Fritillaria*, as sucrose was absent from its nectar. This study also found that sucrose was absence in the nectar of *F. eduardii*. In contrast, the nectar of the very closely related *F. raddeana* contains sucrose and can be described as balanced, based on the ratios of the sugars it contains (fructose, glucose, and sucrose in the ratio 4:3:5). There are large differences in the rate of nectar production within the subgenus *Petilium*. Both *F. imperialis* and *F. eduardii* produce large volumes (204 μl per flower) of dilute nectar (9%), whereas *F. raddeana* produces small volumes (7 μl per flower) of highly concentrated nectar (51%). Theoretically, nectar volumes are under strong selection pressures (e.g., balancing the costs and benefits of nectar production to the plant) and genetic control. However, differences in the volume/sugar concentration of even closely related species have previously been published (Davis et al., 1994).

### Subgenus *Rhinopetalum*

As previously described, the nectaries of this subgenus are furrowed or lobed (Bakhshi Khaniki and Persson, 1997). The aperture of the nectary spur is densely surrounded with short papillae. However, the surface of the nectary area is flat and smooth (Bakhshi Khaniki and Persson, 1997). The presence of the papillae may protect the small quantity of nectar from evaporation or crystallization (Nicolson and Thornburg, 2007). Floral features are influence by ecological factors, like habitat type (Petanidou et al., 2006), and the members of *Rhinopetalum* are normally found in more arid habitats, like semideserts, than is normal within *Fritillaria* (Rix and Zarrei, 2007a,b; Kiani et al., 2017). This study provides the first record of a hexose-rich species (*F. stenanthera*) within the subgenus.

### Subgenus *Theresia*

In both this study and that of Bakhshi Khaniki and Persson (1997), nectaries of *F. persica* were bright and green and contrasted strongly against a dark background (**Figure 12A**). However, Kiani et al. (2017) showed that the appearance of the nectary area as distinct depends on the flower color which is highly variable (pale green, pale yellow, bright yellow, orange, or dark purple), and in some populations, the nectaries of *F. persica* may be difficult to differentiate. Nectaries of the outer tepals are shielded by the inner tepals and are, therefore, probably not easily accessible to visiting insects. SEM analysis revealed that like those of most *Fritillaria* species, the nectaries were uniform and flat and surrounded by an area occupied by slightly convex cells of the tepals (**Figure 12B**). Flowers of *F. persica* produce rather a small amount of nectar, but as a single specimen usually produces several dozen flowers, the overall reward is relatively plentiful. Nectar is strongly hexose-dominant.

### Other Species

Scanning electron microscope analyses and studies of nectar properties were conducted for the first time for *F. grandiflora* and *F. olgae*. The nectaries of *F. grandiflora* and *F. olgae* were similar to those found in flowers of the subgenus *Fritillaria*. Also, SEM analysis revealed the typical *Fritillaria* pattern, comprising a flat and uniform nectary area surrounded by an area bearing convex cells. Both these features might indicate affinities to the subgenus *Fritillaria*. However, *F. olgae* nectar was sucrose-dominant (H:S in the ratio 2:3), such as it is generally found in passerine-pollinated species in the subgenus *Petilium*.

### Ecological Context

The flowers of *Fritillaria* are very diverse – not only in color, shape, and appearance but also in the array of floral rewards like nectar sugar concentration and composition or reward location (Bakhshi Khaniki and Persson, 1997). *Fritillaria* have a wide geographical distribution and occupy a variety of different habitats (Hanson et al., 2009; Kiani et al., 2017). Recent DNA studies show a strong geographic relationship within *Fritillaria* (Day et al., 2014), even among morphologically divergent species. Species rich areas are normally associated with highly variable habitats and/or more recent oscillating climates and microclimates, resulting in numerous range changes, periods of isolation, and recombination (Myers et al., 2000; Kiani et al., 2017). However, some elements of this remarkable diversity might also be the result of a relatively rapid coevolution with their pollinators, as several species, which are distantly related have similar-looking nectaries (convergence): like, for example, *F. pudica* and *F. carica* or *F. purdyi* and *F. crassifolia*, respectively (Rix and Strange, 2014). As many fritillaries are native to remote, difficult to access, or uninhabited areas (Kiani et al., 2017), information regarding their reproduction is limited. Data concerning pollination system or *Fritillaria* flower visitors are only available for six species (Hedström, 1983; Búrquez, 1989; Peters et al., 1995; Minagi et al., 2005; Pendergrass and Robinson, 2005; Zox and Gold, 2008; Zych and Stpiczyńska, 2012; Zych et al., 2014).

In temperate habitats of the northern hemisphere, where *Fritillaria* species grow, most plants are insect-pollinated and are characterized by lack of specialization of their flowers, thus, attracting a large range of insects (Galetto et al., 1998). This is generally the case for *Fritillaria*, where the nectaries are variable and most are easily accessible, therefore, are likely to be visited by a range of different floral visitors. Our microscopical studies revealed that the structures of nectaries of putatively insect pollinated species are similar. Most *Fritillaria* species studied had a relatively flat nectary area surrounded by slightly convex cells, important for insect pollination, providing extra perch during flower manipulation by insects, thus, increasing foraging efficiency (Whitney et al., 2011; Ojeda et al., 2012). Conical papillae, found on the nectaries of *F. pyrenaica*, cause the thin film of the nectar to glisten. In *F. davisii*, the tepal surface and the area adjacent to the nectary was covered with papillae, arranged in rows along the length of the tepal. This may act as a physical nectar guide and a tactile cue, orientating insects toward both the reward and the reproductive parts of the flower. As the floral reward is easily accessible, and the corolla is normally wide open, insects can easily locate and exploit this resource.

Although data from the literature is scare, bees were seen by authors, visiting *Fritillaria* flowers. These animals frequently seek out flowers with medium nectar volumes of medium sugar concentration, often located toward the base of the flower (Willmer, 2011), criteria common in *Fritillaria* flowers. Many *Fritillaria* species have hexose-rich nectar, which according to floral syndrome theory is preferred by short-tongue bees (Chalcoff et al., 2006). However, bee-pollinated plants show a wide range of nectar sugar compositions, as would be expected in the nectar of flowers pollinated by such a large group (Stiles and Freeman, 1993). Pollination by bees is the most common pollinating interaction, and it would be fair to expect that melittophily is the most common syndrome in *Fritillaria*.

Other types of entomogamy are also present in *Fritillaria*, for example, *F. camschatcensis* is fly-pollinated (Zox and Gold, 2008). The checkered pattern found on many flowers of *Fritillaria* might encourage increased visitation by carrion-flies or wasps, with a strong preference for mottled petals. These groups of animals often visit large, tubular flowers with wide-open corollas and dull red, purple, brown, or greenish petals (Willmer, 2011). Several other *Fritillaria* species, such as *F. graeca*, *F. montana*, and *F. davisii* fall into this category. These species produce relatively small volumes of nectar and sometimes emit a disagreeable odor. In *F. camschatcensis* and *F. davisii*, traces of viscous and almost solid nectar form a thick film over the nectary. This would be difficult for pollinators to access. Such presentation of nectar may act as a phenotypic filter, preventing insects other than flies, with have a cushion-like labium, to gather floral rewards (Stpiczyńska et al., 2014).

To date, there is no data on pollinators or floral visitors to members of the subgenus *Rhinopetalum*. They have unusual nectaries concealed in sac-like structures, covered with trichomes, which are not easily accessible. Densely papillose ridges of the nectary apertures potentially exclude feeding animals with relatively short proboscises (Stolar and Davis, 2010) and/or reduce evaporation. All three species examined in this subgenus, produce small volumes of nectar with relatively high sugar concentration. Pale pink or white flowers and nectar concealed in grooves covered with fine hairs are the normal characteristics associated with butterfly pollination, which also occurs in *Lilium martagon*, another species with similar nectaries (Brantjes and Bos, 1980).

Pollinator availability is low for winter or early spring flowering plants as low temperatures impede insect pollinator activity. By contrast, birds, which might be considered ‘alternative pollinators,’ are warm-blooded and more reliable at low temperatures, especially where cold and/or rainy weather conditions might be frequent (Fang et al., 2012). Although several studies indicate that frequent pollinator shifts have occurred during angiosperm speciation events, it may be the case that a large proportion of these events occur relatively late within specific pollination systems (Serrano-Serrano et al., 2017). This might also be the case for *Fritillaria*. Moreover, evidence indicates that the switch from entomophily to ornithophily occurred at least twice during the history of the genus, once for each of the two main clades.

Two very closely related Asian members of the subgenus *Petilium*, *F. imperialis* and *F. eduardii*, fulfill many of the criteria that characterize ornithophilous flowers. They show diurnal anthesis, have scarlet or orange flowers, and lack nectar guides. Their pale anthers and style extend beyond the large corolla, and these robust reproductive elements are able to withstand visits by large pollinators. Although birds do not display innate preference for red (Bené, 1945; Stiles, 1976; Micheneau et al., 2006; Handelman and Kohn, 2014), flowers that are visited by these animals often have red colouration (Goldsmith and Goldsmith, 1979; McDade, 1983; Delph and Lively, 1989). This might suggest that some new characters in bird-pollinated flowers have evolved to discourage visits by illegitimate flower visitors, in this instance insects (Cronk and Ojeda, 2008; Lunau et al., 2011). In *F. imperialis* and *F. eduardii*, the pollen is pale, which makes it less attractive to insects and less prone to potential pollen theft (Wilmsen et al., 2017).

Analysis of nectary morphology revealed the absence of collenchyma, this could have helped the flower to withstand contact with a hard beak, as it occurs in several ornithophilous flowers (Stpiczyńska et al., 2004, 2005, 2009). The starch grains, found in all members of the subgenus *Petilium*, might be regarded as a derived strategy to support the intensive secretion of large amounts of sugar during peak nectary activity (De la Barrera and Nobel, 2004; Heil, 2011; Stpiczyńska et al., 2012). Our studies reveal that this kind of energy storing in members of *Petilium* had two possible results. *Fritillaria* *raddeana* produces small volumes of highly concentrated nectar, whereas *F. eduardii* and *F. imperialis*, on the other hand, produce large volumes of very dilute nectar. In fact, in *F. eduardii*, the concentration of nectar sugar does not even reach 10%. The results for these two species match the data available in the literature, which state that bird-pollinated flowers produce nectar whose low sugar concentration averages 20–25% (w/w) (Nicolson, 2002). This indicates that the attraction of potential bird-pollinators might be important to the various nectar features related to pollination. Moreover, the nectar of *F. imperialis* and *F. eduardii* is hexose-rich, and it lacks even traces of sucrose. As nectar originates from sucrose-rich phloem sap, the proportion of monosaccharides in the final nectar depends on the activity of invertases in the nectary wall. Hydrolysis of sucrose increases the osmolality of the nectar, and the resulting water influx can convert a 30% sucrose nectar into a 20% hexose nectar with a great (1.6 fold) increase in volume. As passerine birds are the largest bird pollinators, they require large amounts of energy and water (Nicolson, 2002).

Different components of nectar respond in different ways to various environmental factors like elevation. Relative sucrose concentration declines in response to increasing elevation, but the percentage of fructose intensifies (Stiles and Freeman, 1993). Usually, this process is gradual, suggesting the response is physiological, possibly temperature related, rather than a reduction in the selection of sucrose-rich nectar. *Fritillaria* *imperialis* grows on rocky slopes at about 1000–3000 m (Tekşen and Aytaç, 2008; Kiani et al., 2017), whereas *F. eduardii* grows at 1200–2100 m and *F. raddeana* grows at 1000 m (Kiani et al., 2017), and the average sucrose concentrations reflect this; the decline in the case of *Petilium* is not gradual. Moreover, species of certain plant families have nectars of relatively consistent sucrose composition (Willmer, 2011), which is also not reflected for *Petilium*. The concentration and composition of nectar varies greatly within this subgenus (*F. imperialis* and *F. eduardii* vs. *F. raddeana*). Nevertheless, the higher hexose content in the nectar of highland plants might originally have facilitated the switch to nectarivory by passerine birds (Stiles, 1978; Stiles and Freeman, 1993), and this may play a significant role in members of *Petilium*. Physiological constraints related to nectar production at higher altitudes may have led to sucrose elimination. Both *F. eduardii* and *F. imperialis* have pendulous, orange or reddish flowers, held on top of a thick stem, which provides a suitable perch for foraging birds. This might potentially lead to further pressure to reduce nectar concentration, which makes flowers less attractive to insect visitors and more attractive to birds, indicating that the nectar properties of *F. imperialis* and *F. eduardii* are the result of double selective pressure.

A second shift to ornithophily occurred in the distinct branch, consisting of mostly American species. Two species, sometimes co-occurring *F. recurva* and *F. gentneri*, also fulfill many of the criteria characteristic of ornithophilous flowers, that is, diurnal anthesis, scarlet flowers lacking nectar guides, and production of copious amounts of rather dilute nectar. However, the flowers of both species are held on a thin, pendulous inflorescence not suitable for perching while feeding but would suit hummingbird pollination (Willmer, 2011). The stamens of *F. recurva* are extended beyond the corolla tube, and, thus, allow contact between the reproductive elements and larger flower visitors. *Fritillaria* *gentneri* is a naturally occurring hybrid between *F. affinis* and *F. recurva*, and it possesses many intermediate flower features, but, without extended stamens. Flowers of *F. gentneri* and *F. recurva* are also visited under natural conditions by andrenids and halictids (Pendergrass and Robinson, 2005). It is likely that they are pollinated both by insects and birds, and we did not find many characters that might discourage illegitimate visitors. Moreover, *F. affinis* is postulated as an insect pollinated species.

Scanning electron microscope analysis revealed that the flat nectary area is surrounded by an area comprising slightly convex tepals cells. This might provide tactile cues for insect pollinators. Bees, for example, prefer such a surface for landing (Whitney et al., 2011). It might also help them to maintain their grip and stay inside the flowers while obtaining nectar from the flat nectary area. The epidermal wall was no thicker than in other species, nor more collenchymatous; it did not seem to provide any extra support and/or protective function, such as preventing damage to the nectary area while coming into contact with the hard beaks (Stpiczyńska et al., 2004, 2005, 2009).

As previously mentioned, nectar of *F. gentneri* and *F. recurva* was more copious and of lower concentration when compared to other closely related species of the subgenus *Liliorhiza*. However, the relative proportion of sugars is similar for all members of the subgenus *Liliorhiza* studied here, and it is characterized by high hexose concentration. Also, nectar of hummingbird-pollinated species is hexose-rich, which is unusual for hummingbird-pollinated flowers. Generally, hummingbirds visit flowers that have sucrose-dominant nectar (Cronk and Ojeda, 2008), also this matches the birds’ recorded preferences in taste tests (Baker, 1975). The intestinal walls of hummingbirds contain a sucrase enzyme, which helps them to tolerate sucrose-rich solutions (del Rio and Karasov, 1990). However, data on hummingbird preferences are often conflicted (del Rio and Karasov, 1990; Lotz and Nicolson, 1996; Willmer, 2011), as these birds freely take hexose-rich nectar when other sources are unavailable (Willmer, 2011). Many flowers visited by hummingbirds are not distinctly adapted to hummingbird-pollination. However, the capacity of hummingbirds to easily extract nectar from open melittophilous flowers, may account for the many shifts toward ornithophily. Moreover, hummingbirds are inquisitive and they investigate many flower types and designs (Wilson et al., 2007), and their spatial memory helps them to return to rewarding plants (Healy and Hurly, 2003). Plants can benefit from these visits, as hummingbirds efficiently transfer pollen even with flowers of a poor morphological fit (Wilson et al., 2007).

Characters found in putative insect-pollinated species of *Fritillaria*, such as rapid nectar replenishment and large, brightly colored corollas, may be regarded as preadaptations for bird-pollination (Wilson et al., 2007). This is, especially, evident in *F. olgae*, a species that produces copious, but rather dilute, sucrose-rich nectar (Castellanos et al., 2003).

Flower features determine which animals or group of animals will be attracted. Moreover, the character and location of the reward can significantly influence the species that are attracted. The relationship between the characteristics of *Fritillaria* nectar and nectaries and their diversity may guide two evolutionary processes: selection of the biotic environment for floral features (sympatric congeners and types of pollinators) and the degree of floral response to this selection (its integration and precision). The attractiveness of these features, on the other hand, ensures that the pollinators attracted to a particular species are affected by these characters. Specialization along this path could result in coevolutionary pollinator attraction or pollinator switches (Armbruster and Muchhala, 2009). From our *Fritillaria* study, the foundation for these switches might be the quantity and quality of the reward offered to flower visitors.

Based on our *Fritillaria* data, such shifts seem unlikely to generate reproductive isolation that would allow sufficient divergence of populations by pollinator selection. Therefore, it is probable that bird-pollinated species of this genus like, for example, *F. imperialis* or *F. recurva* and *F. gentneri* are, and will continue to be, an intermediate phase during which both ancestral and new rewards and advertisements are present, and both ancestral and new pollinators visit the same flower (Armbruster and Muchhala, 2009). There is, still, a considerable need for further studies of *Fritillaria* pollination system in natural habitats and the genetic basis of character shifts in relation to their pollination system.

## Author Contributions

KR and MZ conceived the study and wrote the draft version of the paper. KR, LH, and PK assembled field data. KR, AB, MC, and AG performed the nectar analysis. KR, MZ, JS, MS, and LH analyzed the data. LH and KR assembled photographic documentation. All authors contributed to the final version.

## Conflict of Interest Statement

The authors declare that the research was conducted in the absence of any commercial or financial relationships that could be construed as a potential conflict of interest.

## Footnotes

1 The photographs of flowers and nectaries are representative species for the *Fritillaria* subgenera.
