# Supplementary material for: Functional Diversity of Nectary Structure and Nectar Composition in the Genus Fritillaria (Liliaceae)
Source: Front Plant Sci. 2018 Sep 24;9:1246. doi: 10.3389/fpls.2018.01246 (PMC6187251; doi:10.3389/fpls.2018.01246)
Supplement: Supplementary file 1 [file Table_1.DOCX]

Supplementary Material

**Functional diversity of nectary structure and nectar composition in the genus *Fritillaria* (Liliaceae)**

Katarzyna Roguz^1*^, Andrzej Bajguz^2^, Agnieszka Gołębiewska^2^, Magdalena Chmur^2^, Laurence Hill^3^, Paweł Kalinowski^4^, Jörg Schönenberger^5^, Małgorzata Stpiczyńska^1^, and Marcin Zych^1*^

**Correspondence:**

**k.roguz@biol.uw.edu.pl**

**mzych@biol.uw.edu.pl**


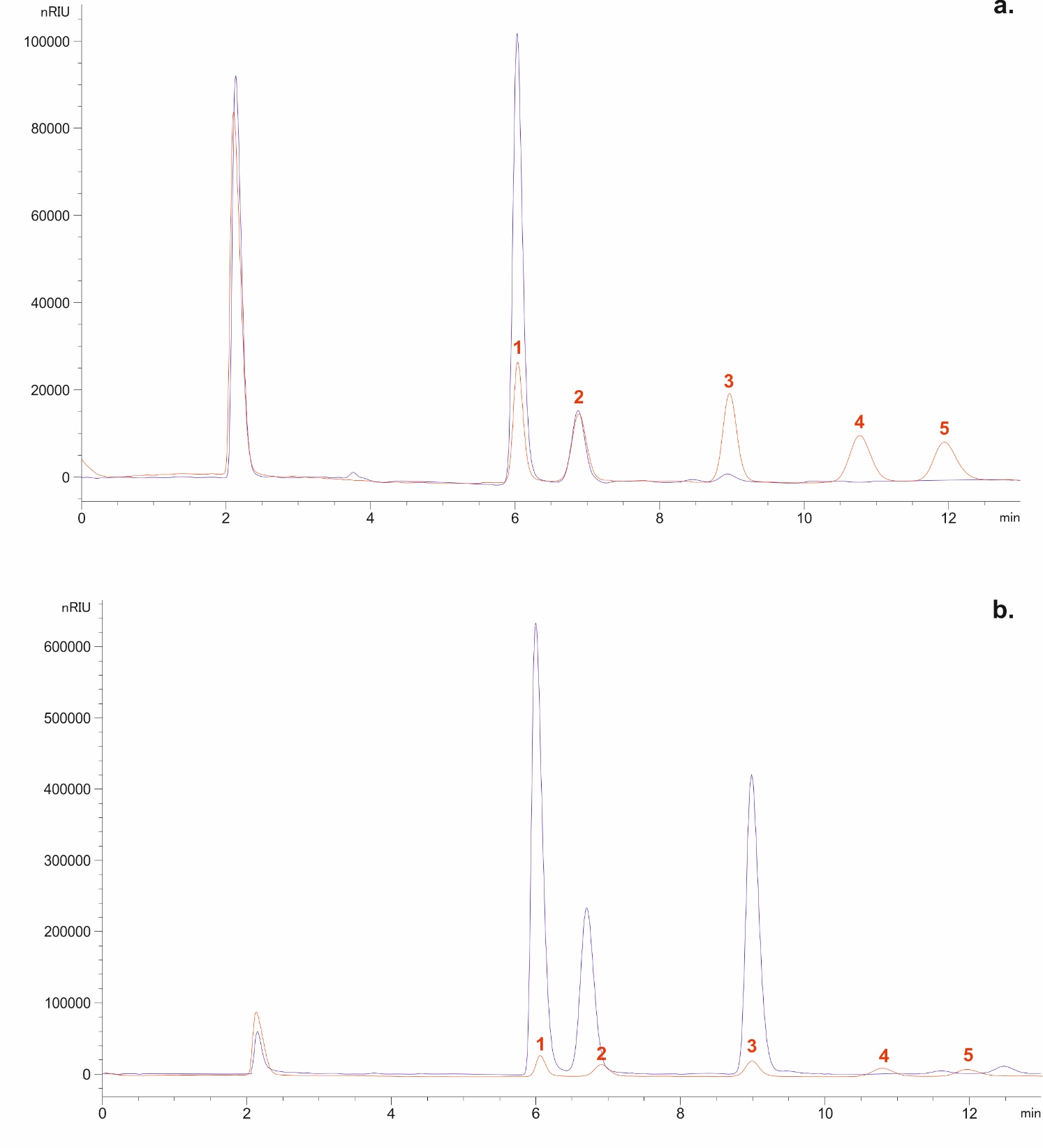


**Supplementary Figure 1.** Separation profile of sugar from nectars of *F. acmopetala* (a) and *F. amabilis* (b) (blue line) compared to reference sugar standards (fructose, 1; glucose, 2; sucrose, 3; maltose, 4; lactose, 5) (red line).


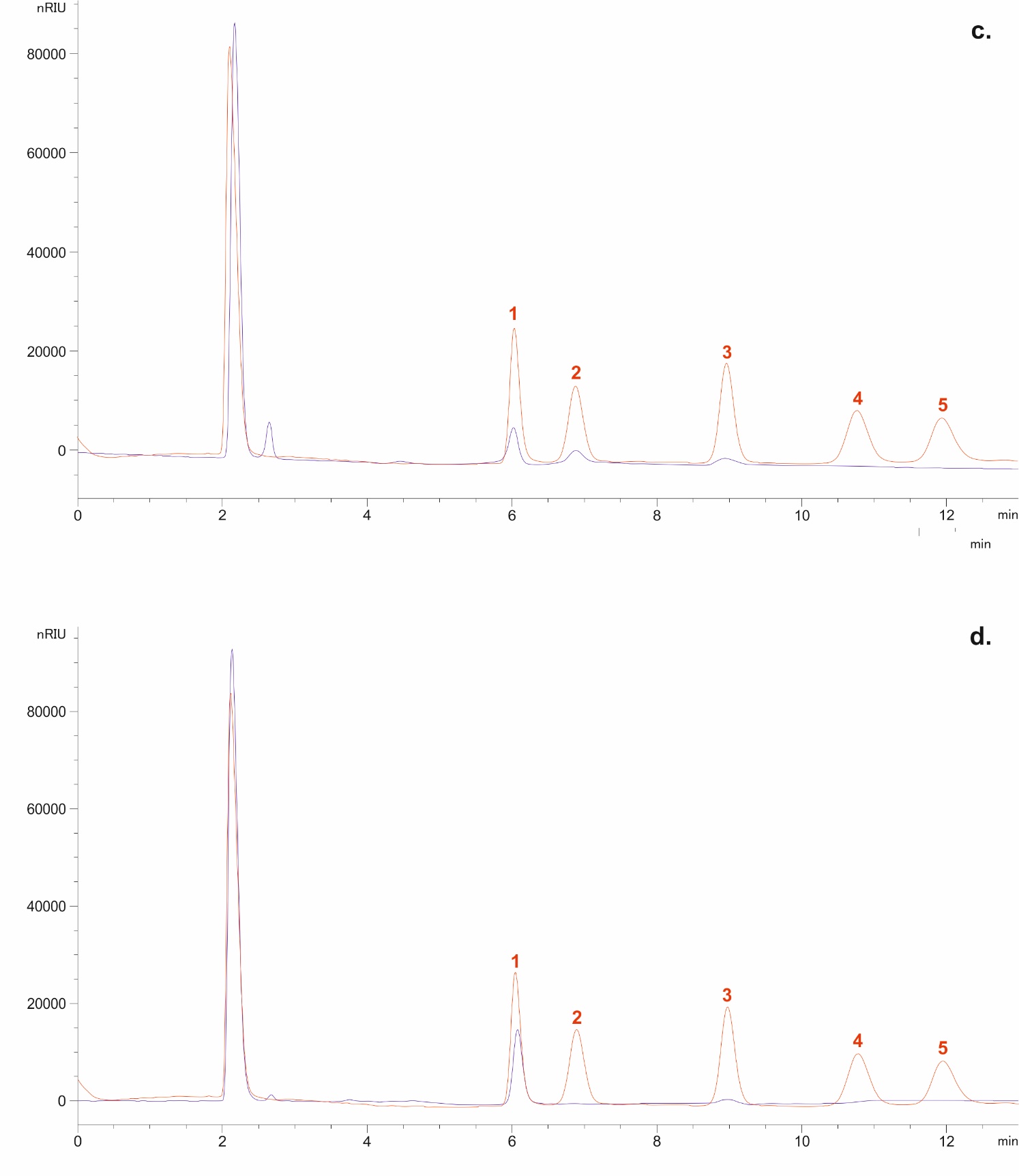


**Supplementary Figure 2.** Separation profile of sugar from nectars of *F. camtschatcensis* (c) and *F. eduardii* (d) (blue line) compared to reference sugar standards (fructose, 1; glucose, 2; sucrose, 3; maltose, 4; lactose, 5) (red line).

**
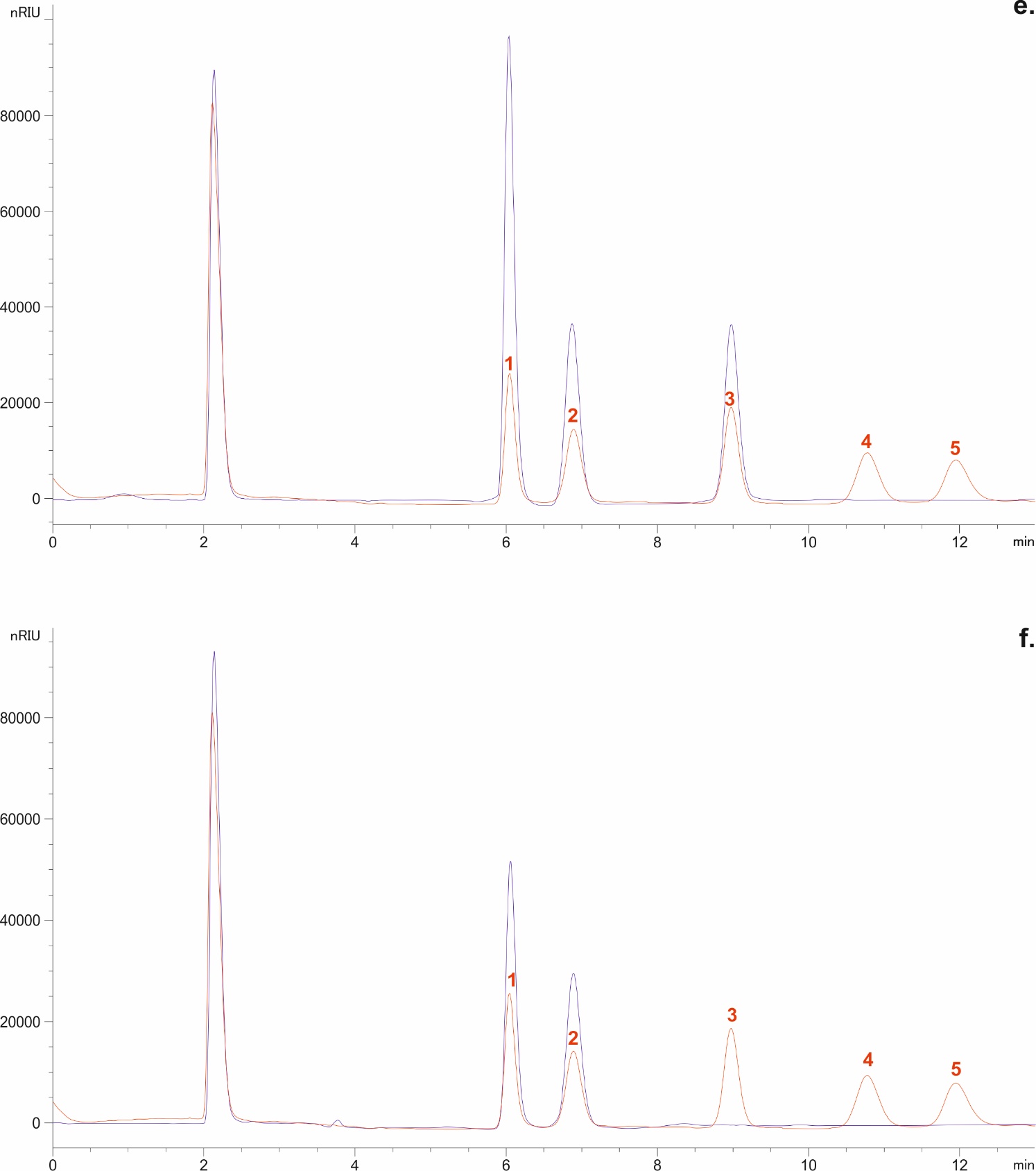
**

**Supplementary Figure 3.** Separation profile of sugar from nectars of *F. gentneri* (e) and *F. imperialis* (f) (blue line) compared to reference sugar standards (fructose, 1; glucose, 2; sucrose, 3; maltose, 4; lactose, 5) (red line).


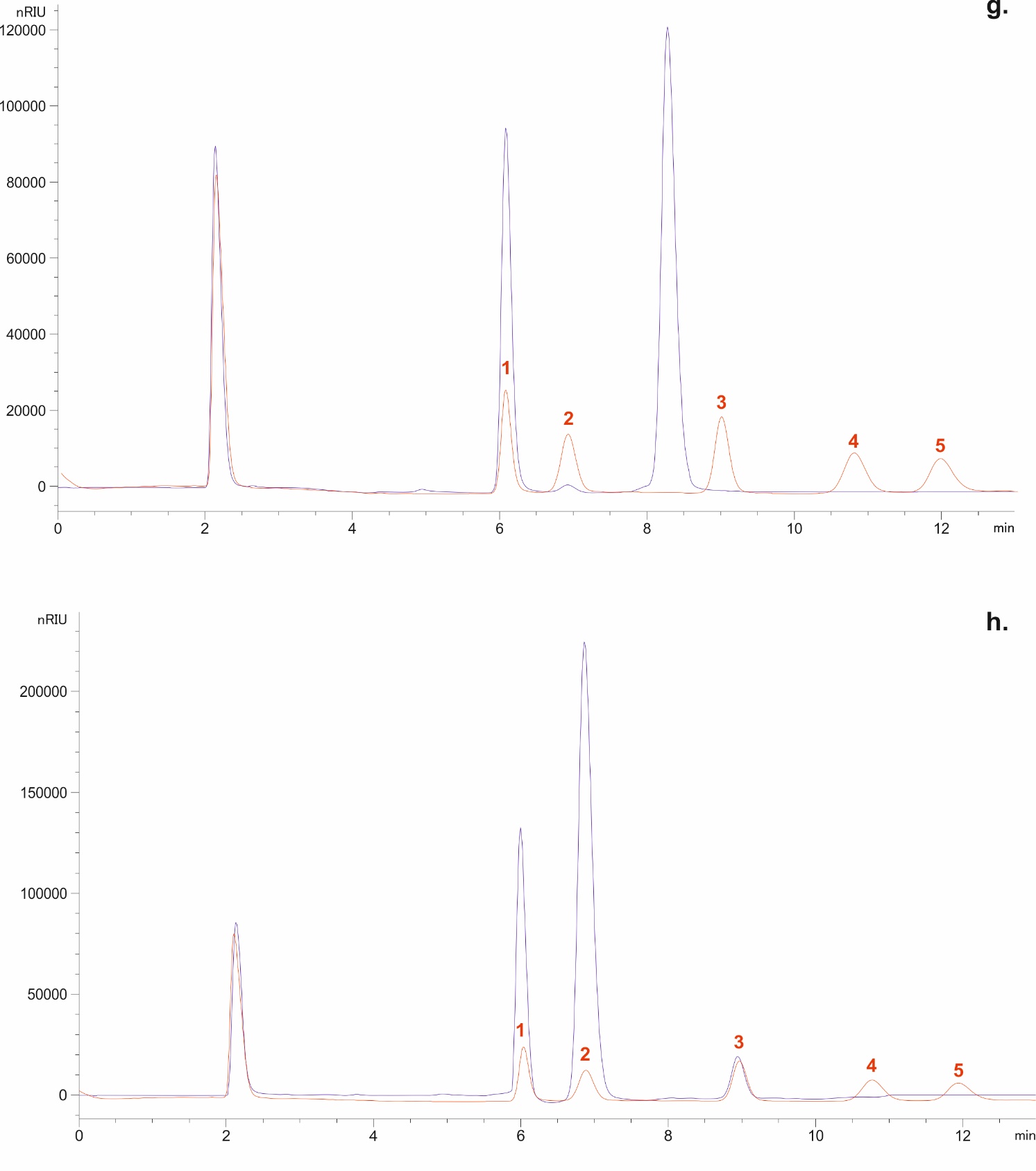


**Supplementary Figure 4.** Separation profile of sugar from nectars of *F. olgae* (g) and *F. persica* (h) (blue line) compared to reference sugar standards (fructose, 1; glucose, 2; sucrose, 3; maltose, 4; lactose, 5) (red line).


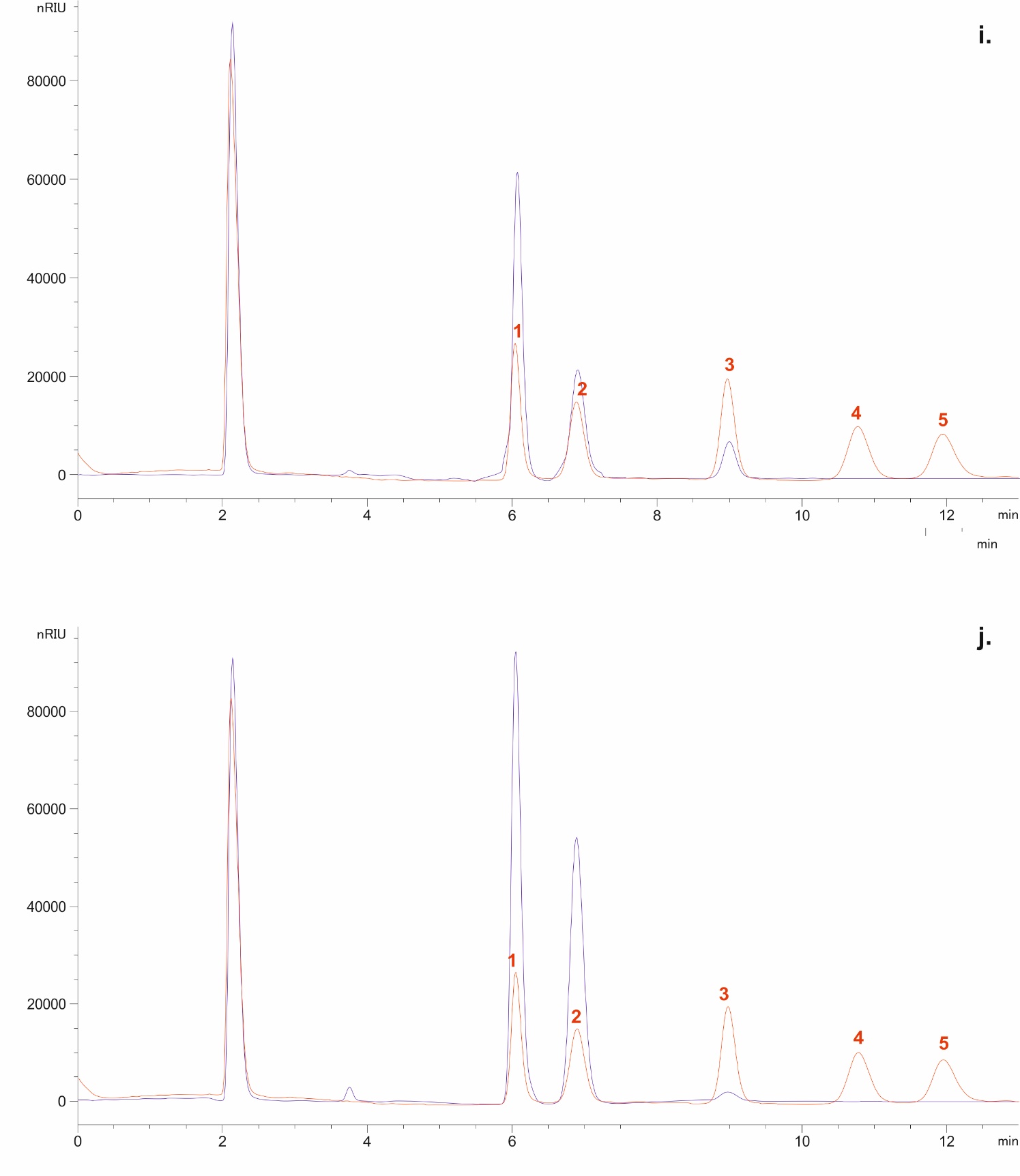


**Supplementary Figure 5.** Separation profile of sugar from nectars of *F. recurva* (i) and *F. sewerzowii* (j) (blue line) compared to reference sugar standards (fructose, 1; glucose, 2; sucrose, 3; maltose, 4; lactose, 5) (red line).


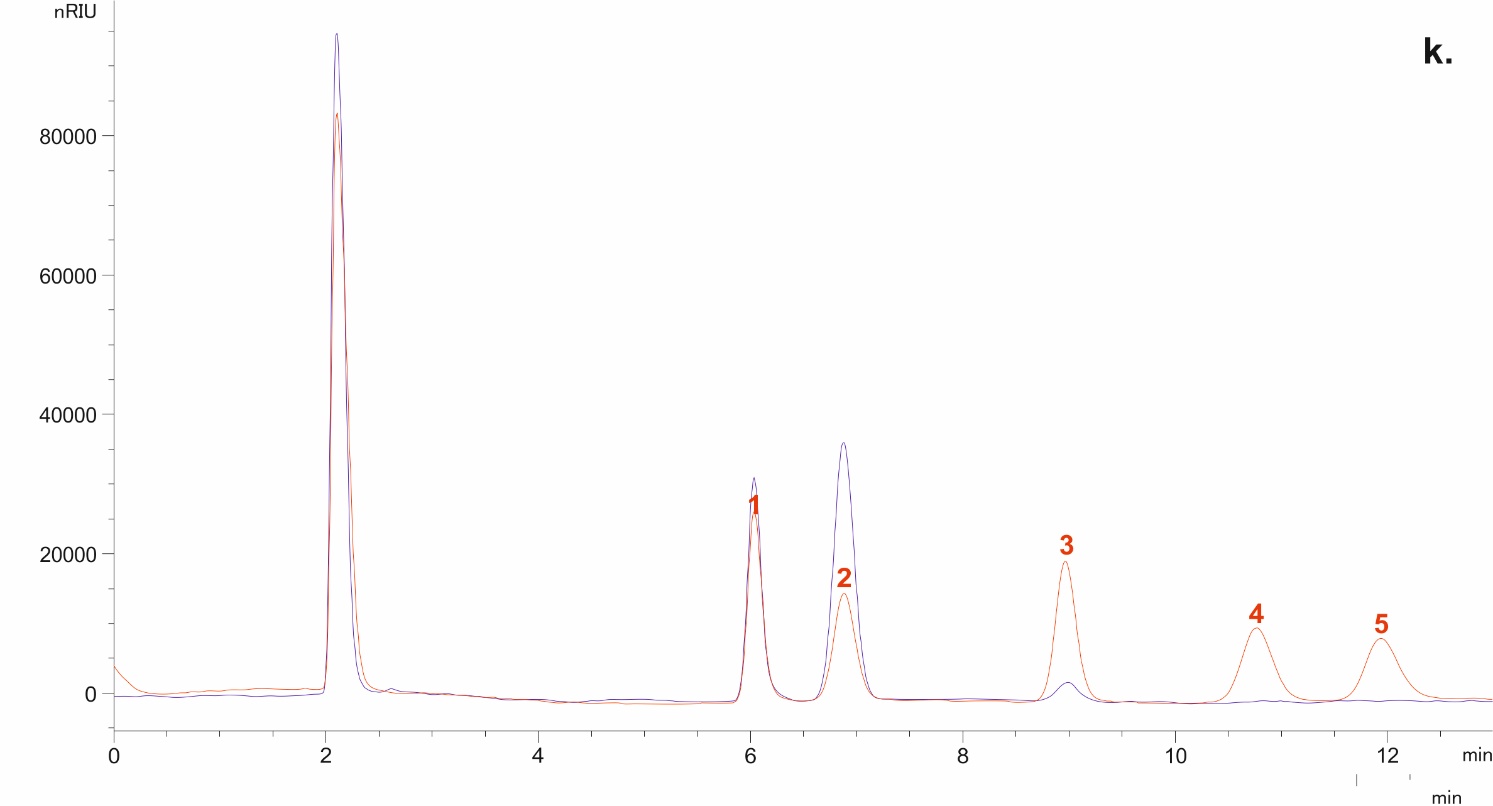


**Supplementary Figure 6.** Separation profile of sugar from nectars of *F. stenanthera* (i) (blue line) compared to reference sugar standards (fructose, 1; glucose, 2; sucrose, 3; maltose, 4; lactose, 5) (red line).
